# Supplementary material for: miRNAs Related to Immune Checkpoint Inhibitor Response: A Systematic Review
Source: Int J Mol Sci. 2024 Feb 1;25(3):1737. doi: 10.3390/ijms25031737 (PMC10855819; doi:10.3390/ijms25031737)
Supplement: Supplementary file 1 [file ijms-25-01737-s001.zip › ijms-2808350-supplementary.pdf]

Supplementary Table S1. List of reviewed works

Title	DOI	Document Type	Year of publication	Analysis
[Genomic Analysis and Immunotherapy by Liquid Biopsy].		Article	2019	Not written in English
[Molecular pathogenesis and its therapeutic implication for ATL]	10.11406/rinketsu.58.2004	Review	2017	Not written in English
[Novel miRNAs as Potential Regulators of PD-1/PD-L1 Immune Checkpoint, and Prognostic Value of MIR9-1 and MIR124-2 Methylation in Ovarian Cancer].	10.31857/S0026898420060075	Article	2020	Not written in English
[Polysaccharide of Atractylodis Macrocephalae Rhizoma inhibits expression of immune checkpoint PD-L1 by targeting miR-34a in esophageal carcinoma cells]	10.19540/j.cnki.cjcmm.20211203.701	Article	2022	Not written in English
[Regulation of PD-L1 by MicroRNA in Mismatch Repair Deficient-Colorectal Cancer].		Article	2017	Not written in English
28P Hijacking CCAT1/miR-17-5p axis alleviates immune checkpoint blockers resistance in PDL1+ TNBC patients	10.1016/j.annonc.2021.01.042	Conference Abstract	2021	Not an original study
3,3 '-Diindolylmethane Ameliorates Staphylococcal Enterotoxin B-Induced Acute Lung Injury through Alterations in the Expression of MicroRNA that Target Apoptosis and Cell-Cycle Arrest in Activated T Cells	10.1124/jpet.115.226563	Article	2016	Not focused on cancer
37P Breast cancer immunopeptidomes reveal a large number of targetable tumor antigens		Conference Abstract	2022	Not an original study
A "Lymphocyte MicroRNA Signature" as Predictive Biomarker of Immunotherapy Response and Plasma PD-1/PD-L1 Expression Levels in Patients with Metastatic Renal Cell Carcinoma: Pointing towards Epiger	10.3390/cancers12113396	Article	2020	Article included in qualitative synthesis
A brand new era of cancer immunotherapy: breakthroughs and challenges	10.1097/CM9.0000000000001490	Review	2021	Not an original study
A Cell Component-Related Prognostic Signature for Head and Neck Squamous Cell Carcinoma Based on the Tumor Microenvironment	10.1155/2022/6022869	Article	2022	Not focused on ICIs
A comparative view on molecular alterations and potential therapeutic strategies for canine oral melanoma	10.3390/vetsci8110286	Review	2021	Not an original study
A Differentiation-Related Gene Prognostic Index Contributes to Prognosis and Immunotherapy Evaluation in Patients with Hepatocellular Carcinoma	10.3390/cells11152302	Article	2022	Not focused on miRNAs
A distinct metabolic response characterizes sensitivity to EZH2 inhibition in multiple myeloma	10.1038/s41419-021-03447-8	Article	2021	Not focused on ICIs
A Ferroptosis-Related Prognostic Risk Score Model to Predict Clinical Significance and Immunogenic Characteristics in Glioblastoma Multiforme	10.1155/2021/9107857	Article	2021	Not focused on miRNAs
A first-in-human study of the anti-LAG-3 antibody favezelimab plus pembrolizumab in previously treated, advanced microsatellite stable colorectal cancer	10.1016/j.esmoop.2022.100639	Article	2022	Not focused on miRNAs
A Glimpse in the Future of Malignant Mesothelioma Treatment	10.3389/fphar.2021.809337	Review	2021	Not an original study
A Holistic Perspective: Exosomes Shuttle between Nerves and Immune Cells in the Tumor Microenvironment	10.3390/jcm9113529	Review	2020	Not an original study
A Lymphocyte MicroRNA Signature as Predictive Biomarker of Immunotherapy Response and Plasma PD-1/PD-L1 Expression Levels in Patients with Metastatic Renal Cell Carcinoma: Pointing towards Epigenetic Reprogramming		Article	2020	Not experimentally demonstrating a direct association between the ICI effect on changes in the levels of miRNAs or vice versa
A novel immune resistance mechanism of melanoma cells controlled by the ADAR1 enzyme	10.18632/oncotarget.4905	Article	2015	Not experimentally demonstrating a direct association between the ICI effect on changes in the levels of miRNAs or vice versa
A Novel m6A-Related LncRNA Signature for Predicting Prognosis, Chemotherapy and Immunotherapy Response in Patients with Lung Adenocarcinoma		Article	2022	Not experimentally demonstrating a direct association between the ICI effect on changes in the levels of miRNAs or vice versa
A Novel P53 Nanomedicine Reduces Immunosuppression and Augments Anti-PD-1 Therapy for Non-Small Cell Lung Cancer in Syngeneic Mouse Models	10.3390/cells11213434	Article	2022	Not experimentally demonstrating a direct association between the ICI effect on changes in the levels of miRNAs or vice versa
A novel risk prediction model of pyroptosis-related genes for the prognosis and immunotherapy response of endometrial cancer	10.26355/eurrev_202204_28456	Article	2022	Full text unavalaible
A panel of seven immune-related genes can serve as a good predictive biomarker for cervical squamous cell carcinoma	10.3389/fgene.2022.1024508	Article	2022	Not focused on miRNAs
A phase 2 trial of trifluridine/tipiracil plus nivolumab in patients with heavily pretreated microsatellite-stable metastatic colorectal cancer	10.1002/cam4.3630	Article	2021	Not focused on miRNAs
A Pilot Study Comparing the Efficacy of Lactate Dehydrogenase Levels Versus Circulating Cell-Free microRNAs in Monitoring Responses to Checkpoint Inhibitor Immunotherapy in Metastatic Melanoma Patient	10.3390/cancers12113361	Article	2020	Article included in qualitative synthesis
A scoping review on the potentiality of PD-L1-inhibiting microRNAs in treating colorectal cancer: Toward single-cell sequencing-guided biocompatible-based delivery	10.1016/j.biopha.2021.112213	Review	2021	Not an original study
A six-miRNA signature as a novel biomarker for improving prediction of prognosis and patterns of immune infiltration in hepatocellular carcinoma		Article	2022	Not focused on ICIs
A Systematic Review on the Therapeutic Potentiality of PD-L1-Inhibiting MicroRNAs for Triple-Negative Breast Cancer: Toward Single-Cell Sequencing-Guided Biomimetic Delivery	10.3390/genes12081206	Review	2021	Not an original study
A Transcription Factor Signature Can Identify the CMS4 Subtype and Stratify the Prognostic Risk of Colorectal Cancer	10.3389/fonc.2022.902974	Article	2022	Not focused on miRNAs
ACKR4 in tumor cells regulates dendritic cell migration to tumor-draining lymph nodes and T-cell priming	10.3390/cancers13195021	Article	2021	Not experimentally demonstrating a direct association between the ICI effect on changes in the levels of miRNAs or vice versa
ADRENERGIC SIGNALING AUGMENTS CYTOTOXIC T CELL ANTI TUMOR ACTIVITY IN P53 DEFICIENT CANCERS BY CXCL10 RELEASE	10.1136/jitc-2022-SITC2022.1462	Conference Abstract	2022	Not an original study
Adrenergic signaling augments cytotoxic T-cell anti-tumor function in p53-deficient head and neck cancer		Conference Abstract	2021	Not an original study
Advanced penile cancer presenting with renal failure	10.46883/ONC.2021.3507.0425	Article	2021	Not focused on miRNAs
Advances in Biomarkers for Detecting Early Cancer Treatment-Related Cardiac Dysfunction	10.3389/fcvm.2021.753313	Review	2021	Not an original study
Advances in Human Immune System Mouse Models for Personalized Treg-Based Immunotherapies	10.3389/fimmu.2021.643544	Review	2021	Not an original study
Advances in Next-Generation Sequencing Bioinformatics for Clinical Diagnostics: Taking Precision Oncology to the Next Level	10.1016/j.yamp.2018.06.010	Review	2018	Not an original study
Against the motion: Treatment intensification in stage III NSCLC is a dream rather than a reality?		Conference Abstract	2018	Not an original study
Altered Expression of Epigenetic Modifiers Identifies Novel Biomarkers and Therapeutic Targets in AL Amyloidosis	10.1182/blood-2021-153532	Meeting Abstract	2021	Not an original study
An exosomal miRNA signature as predictor of benefit from immune checkpoint inhibitors in non-small cell lung cancer	10.1016/j.annonc.2020.08.1591	Meeting Abstract	2020	Not an original study
An exosomal mirna signature to predict outcomes of non-small cell lung cancer patients during treatment with nivolumab	10.1177/0300891620953388	Conference Abstract	2020	Not an original study
An immune relevant signature for predicting prognoses and immunotherapeutic responses in patients with muscle-invasive bladder cancer (MIBC)	10.1002/cam4.2942	Article	2020	Not experimentally demonstrating a direct association between the ICI effect on changes in the levels of miRNAs or vice versa
An Integrated Immune-Related Bioinformatics Analysis in Glioma: Prognostic Signature's Identification and Multi-Omics Mechanisms' Exploration	10.3389/fgene.2022.889629	Article	2022	Not focused on ICIs
An Update on the Molecular and Clinical Characteristics of Apocrine Carcinoma of the Breast	10.1016/j.clbc.2021.12.009	Review	2022	Not an original study
Anti-tumor Drug Targets Analysis: Current Insight and Future Prospect	10.2174/1389450120666190402145325	Review	2019	Not an original study
Antitumor immunity is defective in T cell-specific microRNA-155-deficient mice and is rescued by immune checkpoint blockade	10.1074/jbc.M117.808121	Article	2017	Article included in qualitative synthesis
Apatinib combined with Keytruda treatment induces apoptosis of gastric carcinoma cells through CES4/miR-616-5p/DUSP2 axis	10.1111/bcpt.13641	Article	2021	Not experimentally demonstrating a direct association between the ICI effect on changes in the levels of miRNAs or vice versa
ARNTL2 is an indicator of poor prognosis, promotes epithelial-to-mesenchymal transition and inhibits ferroptosis in lung adenocarcinoma	10.1016/j.tranon.2022.101562	Article	2022	Not focused on miRNAs
Assessment and Management of Cardiotoxicity in Hematologic Malignancies	10.1155/2021/6616265	Review	2021	Not an original study
Association of Circular RNA and Long Non-Coding RNA Dysregulation with the Clinical Response to Immune Checkpoint Blockade in Cutaneous Metastatic Melanoma	10.3390/biomedicines10102419	Article	2022	Not experimentally demonstrating a direct association between the ICI effect on changes in the levels of miRNAs or vice versa
Autoimmune Complications in Hematologic Neoplasms	10.3390/cancers13071532	Review	2021	Not an original study
Autoimmunity in Acute Myocarditis: How Immunopathogenesis Steers New Directions for Diagnosis and Treatment	10.1007/s11886-020-01278-1	Review	2020	Not an original study
Autophagy blockade potentiates cancer-associated immunosuppression through programmed death ligand-1 upregulation in bladder cancer	10.1002/jcp.30817	Article	2022	Not experimentally demonstrating a direct association between the ICI effect on changes in the levels of miRNAs or vice versa
Biliary Tract Cancers: Treatment Updates and Future Directions in the Era of Precision Medicine and Immuno-Oncology	10.3389/fonc.2021.768009	Review	2021	Not an original study
Bioinformatics-Based Analysis: Noncoding RNA-Mediated COL10A1 Is Associated with Poor Prognosis and Immune Cell Infiltration in Pancreatic Cancer	10.1155/2022/7904982	Article	2022	Not focused on ICIs
Biological effects of COVID-19 on lung cancer: Can we drive our decisions	10.3389/fonc.2022.1029830	Review	2022	Not an original study
Biomarkers for hepatocellular carcinoma: What's new on the horizon?	10.3748/wjg.v24.i35.3974	Review	2018	Not an original study
Biomarkers in Immunotherapy-Based Precision Treatments of Digestive System Tumors	10.3389/fonc.2021.650481	Review	2021	Not an original study
Blocking IL-17A enhances tumor response to anti-PD-1 immunotherapy in microsatellite stable colorectal cancer	10.1136/jitc-2020-001895	Article	2021	Article included in qualitative synthesis
Blood-based miRNA, exosomal PD-L1, and immune profiling analysis as predictive biomarkers for immune checkpoint inhibitor treatment response in urothelial cancer	10.1200/JCO.2022.40.16_suppl.e16559	Conference Abstract	2022	Not an original study
Bone and soft tissue tumors: New treatment approaches	10.3390/cancers13081832	Editorial	2021	Not an original study
Breast cancer cell–derived microRNA-155 suppresses tumor progression via enhancing immune cell recruitment and antitumor function	10.1172/JCI157248	Article	2022	Article included in qualitative synthesis
CA9-Related Acidic Microenvironment Mediates CD8+ T Cell Related Immunosuppression in Pancreatic Cancer	10.3389/fonc.2021.832315	Article	2022	Not focused on miRNAs
Cancer immunotherapy by blocking immune checkpoints on innate lymphocytes	10.3390/cancers12123504	Review	2020	Not an original study
CAR-T cell combination therapy: the next revolution in cancer treatment	10.1186/s12935-022-02778-6	Review	2022	Not an original study
Cardiac Biomarkers in Patients with Cancer: Considerations, Clinical Implications, and Future Avenues	10.1007/s11912-020-00930-x	Review	2020	Not an original study
Cardiovascular toxic effects of antitumor agents: Pathogenetic mechanisms	10.1016/j.thromres.2021.12.017	Article	2022	Not experimentally demonstrating a direct association between the ICI effect on changes in the levels of miRNAs or vice versa
CASC1 Expression in Bladder Cancer Is Regulated by Exosomal miRNA-150: A Comprehensive Pan-Cancer and Bioinformatics Study	10.1155/2022/8100325	Article	2022	Not experimentally demonstrating a direct association between the ICI effect on changes in the levels of miRNAs or vice versa
CDK7 Inhibition Potentiates Genome Instability Triggering Anti-tumor Immunity in Small Cell Lung Cancer		Article	2020	Not focused on miRNAs
Challenges for better diagnosis and management of pancreatic and biliary tract cancers focusing on blood biomarkers: A systematic review	10.3390/cancers13164220	Review	2021	Not an original study
Characterization of cancer genomic heterogeneity by next-generation sequencing advances precision medicine in cancer treatment	10.1093/pcmedi/pby007	Review	2018	Not an original study
Characterization of the Prognostic m6A-Related lncRNA Signature in Gastric Cancer	10.3389/fonc.2021.630260	Article	2021	Not focused on miRNAs
Cholangiocarcinoma: The role of genetic and epigenetic factors; current and prospective treatment with checkpoint inhibitors and immunotherapy		Review	2021	Not an original study
CircRNA-Based Cervical Cancer Prognosis Model, Immunological Validation and Drug Prediction	10.3390/curroncol29110633	Article	2022	Not experimentally demonstrating a direct association between the ICI effect on changes in the levels of miRNAs or vice versa
Circular RNA CELF1 drives immunosuppression and anti-PD1 therapy resistance in non-small cell lung cancer via the miR-491-5p/EGFR axis	10.18632/aging.203576	Article	2021	Not experimentally demonstrating a direct association between the ICI effect on changes in the levels of miRNAs or vice versa
Circular RNA circMET drives immunosuppression and anti-PD1 therapy resistance in hepatocellular carcinoma via the miR-30-5p/snail/DPP4 axis	10.1186/s12943-020-01213-6	Article	2020	Not experimentally demonstrating a direct association between the ICI effect on changes in the levels of miRNAs or vice versa
Circular rna hsa_circ_0000190 facilitates the tumorigenesis and immune evasion by upregulating the expression of soluble pd-l1 in non-small-cell lung cancer	10.3390/ijms23010064	Article	2022	Not focused on miRNAs
Circulating Biomarkers of Response and Toxicity of Immunotherapy in Advanced Non-Small Cell Lung Cancer (NSCLC): A Comprehensive Review	10.3390/cancers13081794	Review	2021	Not an original study
Circulating microRNAs associated with prolonged overall survival in lung cancer patients treated with nivolumab	10.1080/0284186X.2018.1465585	Article	2018	Not experimentally demonstrating a direct association between the ICI effect on changes in the levels of miRNAs or vice versa
Circulating microRNAs predict the response to anti-PD-1 therapy in non-small cell lung cancer	10.1016/j.ygeno.2019.11.019	Article	2020	Not experimentally demonstrating a direct association between the ICI effect on changes in the levels of miRNAs or vice versa
Circulating miRNAs and PD-L1 Tumor Expression Are Associated with Survival in Advanced NSCLC Patients Treated with Immunotherapy: a Prospective Study	10.1158/1078-0432.CCR-18-1981	Article	2019	Not experimentally demonstrating a direct association between the ICI effect on changes in the levels of miRNAs or vice versa
Cisplatin-induced immune modulation in ovarian cancer mouse models with distinct inflammation profiles		Article	2019	Not focused on miRNAs
Clinical immunology and immunotherapy for hepatocellular carcinoma: current progress and challenges	10.1007/s12072-019-09967-y	Review	2019	Not an original study
Clinicopathologic implications of CD8(+)/Foxp3(+) ratio and miR-574-3p/PD-L1 axis in spinal chordoma patients		Article	2018	Not experimentally demonstrating a direct association between the ICI effect on changes in the levels of miRNAs or vice versa
Combination Immunotherapy for Hepatocellular Carcinoma: Where Are We Currently?	10.1055/s-0040-1722646	Article	2021	Full text unavalaible
Combining Radiation Therapy with Immune Checkpoint Blockade for Central Nervous System Malignancies		Review	2016	Not an original study
COMPLEMENTing immunotherapy	10.1038/s43018-022-00442-6	Review	2022	Not an original study
Comprehensive Analyses of MELK-Associated ceRNA Networks Reveal a Potential Biomarker for Predicting Poor Prognosis and Immunotherapy Efficacy in Hepatocellular Carcinoma	10.3389/fcell.2022.824938	Article	2022	Not focused on the miRNA/ICI relationship
Comprehensive Analysis of Correlations in the Expression of miRNA Genes and Immune Checkpoint Genes in Bladder Cancer Cells	10.3390/ijms22052553	Article	2021	Not experimentally demonstrating a direct association between the ICI effect on changes in the levels of miRNAs or vice versa
Comprehensive Analysis of m6A RNA Methylation Regulators and the Immune Microenvironment to Aid Immunotherapy in Pancreatic Cancer	10.3389/fimmu.2021.769425	Article	2021	Not focused on miRNAs
Comprehensive analysis of the relationship between competitive endogenous RNA (ceRNA) networks and tumor infiltrating-cells in hepatocellular carcinoma		Article	2020	Not experimentally demonstrating a direct association between the ICI effect on changes in the levels of miRNAs or vice versa
Comprehensive analysis of tumor immune microenvironment and prognosis of m6A-related lncRNAs in gastric cancer	10.1186/s12885-022-09377-8	Article	2022	Not experimentally demonstrating a direct association between the ICI effect on changes in the levels of miRNAs or vice versa
Comprehensive Analysis of VCAN Expression Profiles and Prognostic Values in HCC	10.3389/fgene.2022.900306	Article	2022	Not experimentally demonstrating a direct association between the ICI effect on changes in the levels of miRNAs or vice versa
Comprehensive and Integrative Genomic Characterization of Hepatocellular Carcinoma	10.1016/j.cell.2017.05.046	Article	2017	Not focused on the miRNA/ICI relationship
Comprehensive bioinformatics analysis of ribonucleoside diphosphate reductase subunit M2(RRM2) gene correlates with prognosis and tumor immunotherapy in pan-cancer	10.18632/aging.204315	Article	2022	Not experimentally demonstrating a direct association between the ICI effect on changes in the levels of miRNAs or vice versa
Comprehensive Genomic Characterization of Tumor Microenvironment and Relevant Signature in Clear Cell Renal Cell Carcinoma	10.3389/fonc.2022.749119	Article	2022	Not experimentally demonstrating a direct association between the ICI effect on changes in the levels of miRNAs or vice versa
Comprehensive Transcriptome and Pathway Analyses Revealed Central Role for Fascin in Promoting Triple-Negative Breast Cancer Progression	10.3390/ph14121228	Article	2021	Not focused on ICIs
Conjunctival melanoma: New insights in tumour genetics and immunology, leading to new therapeutic options	10.1016/j.preteyeres.2021.100971	Review	2022	Not an original study
Conjunctival melanoma: Update on genetics, epigenetics and targeted molecular and immune-based therapies	10.2147/OPTH.S271569	Review	2020	Not an original study
Construction of a ceRNA network in hepatocellular carcinoma and comprehensive analysis of immune infiltration patterns		Article	2021	Not experimentally demonstrating a direct association between the ICI effect on changes in the levels of miRNAs or vice versa
Construction of a paclitaxel-related competitive endogenous RNA network and identification of a potential regulatory axis in pancreatic cancer	10.1016/j.tranon.2022.101419	Article	2022	Not experimentally demonstrating a direct association between the ICI effect on changes in the levels of miRNAs or vice versa
Correction: Metabolism and epigenetic interplay in cancer: Regulation and putative therapeutic targets (Frontiers in Genetics (2019) DOI: 10.3389/fgene.2018.00427)	10.3389/fgene.2019.00784	Article	2019	Not focused on ICIs
Correlation of plasma exosomal microRNAs with the efficacy of immunotherapy in EGFR/ALK wild-type advanced non-small cell lung cancer	10.1136/jitc-2019-000376	Article	2020	Not experimentally demonstrating a direct association between the ICI effect on changes in the levels of miRNAs or vice versa
Crosstalk between non-coding RNAs expression profile, drug resistance and immune response in breast cancer	10.1016/j.phrs.2021.106041	Review	2022	Not an original study
CTNNB1 mutation suppresses infiltration of immune cells in hepatocellular carcinoma through miRNA-mediated regulation of chemokine expression	10.1016/j.intimp.2020.107043	Article	2020	Not experimentally demonstrating a direct association between the ICI effect on changes in the levels of miRNAs or vice versa
Current and Future Perspectives of PD-1/PDL-1 Blockade in Cancer Immunotherapy	10.1155/2021/6661406	Review	2021	Not an original study
Current concepts of non-coding RNA regulation of immune checkpoints in cancer	10.1016/j.mam.2019.09.007	Review	2019	Not an original study
Cutaneous Angiosarcomas: Molecular Pathogenesis Guides Novel Therapeutic Approaches	10.1111/ddg.14694	Review	2022	Not an original study
Data-driven miRNA classifier as response predictor for immune checkpoint inhibitor treatment in advanced-stage cancer patients	10.1093/annonc/mdz438.004	Conference Abstract	2019	Not an original study
Dendritic Cell-Derived exosomes increase the efficacy of anti-PD-L1 antibody in melanoma model	10.1111/cas.13904	Conference Abstract	2018	Not an original study
Determinants of resistance to VEGF-TKI and immune checkpoint inhibitors in metastatic renal cell carcinoma	10.1186/s13046-021-01961-3	Review	2021	Not an original study
Development and Validation of a Robust Ferroptosis-Related Gene Panel for Breast Cancer Disease-Specific Survival	10.3389/fcell.2021.709180	Article	2021	Not focused on the miRNA/ICI relationship
Development of a Novel Immune Infiltration-Based Gene Signature to Predict Prognosis and Immunotherapy Response of Patients With Cervical Cancer	10.3389/fimmu.2021.709493	Article	2021	Not focused on miRNAs
Development of a potential biomarker for PDL1 expression in lung carcinoma	10.1111/resp.14150	Conference Abstract	2021	Not an original study
Development of a Prognostic Model Based on Pyroptosis-Related Genes in Pancreatic Adenocarcinoma	10.1155/2022/9141117	Article	2022	Not focused on the miRNA/ICI relationship
Development of group b coxsackievirus as an oncolytic virus: Opportunities and challenges	10.3390/v13061082	Review	2021	Not an original study
Diagnostic and therapeutic applications of miRNA-based strategies to cancer immunotherapy		Review	2018	Not an original study
Disentangling the therapeutic tactics in GBM: From bench to bedside and beyond	10.1002/cbin.11484	Review	2021	Not an original study
Disorder of G2-M Checkpoint Control in Aniline-Induced Cell Proliferation in Rat Spleen	10.1371/journal.pone.0131457	Article	2015	Not focused on ICIs
Disruption of Cell-Cell Communication in Anaplastic Thyroid Cancer as an Immunotherapeutic Opportunity	10.1007/978-3-030-83282-7_2	Chapter	2021	Not an original study
DNMT1 as a therapeutic target in pancreatic cancer: mechanisms and clinical implications	10.1007/s13402-020-00526-4	Review	2020	Not an original study
DNMT1: A key drug target in triple-negative breast cancer	10.1016/j.semcancer.2020.05.010	Review	2021	Not an original study
Docosahexaenoic acid may inhibit immune evasion of colorectal cancer cells through targeting immune checkpoint and immunomodulator genes and their controlling microRNAs	10.1002/biof.1842	Article	2022	Not experimentally demonstrating a direct association between the ICI effect on changes in the levels of miRNAs or vice versa
Downregulation of ZC3H13 by miR-362-3p/miR-425-5p is associated with a poor prognosis and adverse outcomes in hepatocellular carcinoma	10.18632/aging.203939	Article	2022	Not experimentally demonstrating a direct association between the ICI effect on changes in the levels of miRNAs or vice versa
Drivers of Gene Expression Dysregulation in Pancreatic Cancer	10.1016/j.trecan.2021.01.008	Review	2021	Not an original study
EBAG9 silencing exerts an immune checkpoint function without aggravating adverse effects	10.1016/j.ymthe.2022.07.009	Article	2022	Not focused on the miRNA/ICI relationship
EBV-associated gastric cancer evades T-cell immunity by PD-1/PD-L1 interactions	10.1007/s10120-018-0880-4	Article	2019	Not experimentally demonstrating a direct association between the ICI effect on changes in the levels of miRNAs or vice versa
Editorial: Biomarker Detection Algorithms and Tools for Medical Imaging or Omics Data	10.3389/fgene.2022.919390	Editorial	2022	Not an original study
Editorial: Biomarkers of Immune-Checkpoint-Inhibitor Immunotherapies in Hepatocellular Carcinomas	10.3389/fonc.2022.860964	Editorial	2022	Not an original study
Editorial: Cancer evolution: From biological insights to therapeutic opportunities	10.3389/fgene.2022.984032	Editorial	2022	Not an original study
Editorial: Clinical and pathogenic novelties in cholangiopathies: New treatments and many more to come	10.1097/MOG.0000000000000804	Editorial	2022	Not an original study
Editorial: DNA Methylation Dynamics and Human Diseases	10.3389/fcell.2022.956286	Editorial	2022	Not an original study
Editorial: Eicosanoids in Cancer	10.3389/fphar.2021.765214	Editorial	2021	Not an original study
Editorial: Emerging Biomarkers for NSCLC: Recent Advances in Diagnosis and Therapy	10.3389/fonc.2021.694578	Editorial	2021	Not an original study
Editorial: Genetic and Epigenetic Control of Immune Responses	10.3389/fimmu.2021.775101	Editorial	2021	Not an original study
Editorial: Head & Neck Cancer and Esophageal Cancer: From Biosignatures to Therapeutics	10.3389/fonc.2021.666103	Editorial	2021	Not an original study
Editorial: Role of epigenetic regulators in the initiation, progression, and metastasis of cancer	10.3389/fgene.2022.978097	Editorial Material	2022	Not an original study
Editorial: Therapeutic Opportunities and Innovative Biomarkers in Tumor Microenvironment	10.3389/fonc.2021.803414	Editorial	2021	Not an original study
Editorial: Tumor microenvironment signaling networks in pathophysiology and therapeutics	10.3389/fonc.2022.1009187	Editorial	2022	Not an original study
Effect of radiotherapy on T cell and PD-1 / PD-L1 blocking therapy in tumor microenvironment	10.1080/21645515.2020.1840254	Review	2021	Not an original study
Effects of helicobacter pylori on tumor microenvironment and immunotherapy responses	10.3389/fimmu.2022.923477	Review	2022	Not an original study
Effects of microbiota on anticancer drugs: Current knowledge and potential applications	10.1016/j.ebiom.2022.104197	Review	2022	Not an original study
Effects of microenvironment in osteosarcoma on chemoresistance and the promise of immunotherapy as an osteosarcoma therapeutic modality	10.3389/fimmu.2022.871076	Review	2022	Not an original study
Efficacy of pembrolizumab in patients with advanced cancer of unknown primary (CUP): a phase 2 non-randomized clinical trial	10.1136/jitc-2022-004822	Article	2022	Not focused on miRNAs
EGFR signaling pathway as therapeutic target in human cancers	10.1016/j.semcancer.2022.04.002	Review	2022	Not an original study
Emerging Blood-Based Biomarkers for Predicting Immunotherapy Response in NSCLC	10.3390/cancers14112626	Review	2022	Not an original study

Emerging Challenges of Radiation-Associated Cardiovascular Dysfunction (RACVD) in Modern Radiation Oncology: Clinical Practice, Bench Investigation, and Multidisciplinary Care	10.3389/fcvm.2020.00016	Review	2020	Not an original study
Emerging drugs for the treatment of cutaneous T-cell lymphoma	10.1080/14728214.2022.2049233	Review	2022	Not an original study
Emerging role of extracellular vesicles in immune regulation and cancer progression	10.3390/cancers12123563	Review	2020	Not an original study
Emerging Role of Non-Coding RNAs in Regulation of T-Lymphocyte Function	10.3389/fimmu.2021.756042	Review	2021	Not an original study
Emerging role of RNA interference in immune cells engineering and its therapeutic synergism in immunotherapy		Review	2021	Not an original study
Emerging Therapeutic Targets in Chordomas: A Review of the Literature in the Genomic Era	10.1093/neuros/nyz342	Review	2020	Not an original study
Engineered extracellular vesicles: potentials in cancer combination therapy	10.1186/s12951-022-01330-y	Review	2022	Not an original study
Epi-immunotherapy for cancers: rationales of epi-drugs in combination with immunotherapy and advances in clinical trials	10.1002/cac2.12313	Review	2022	Not an original study
Epigenetic Mechanisms of Resistance to Immune Checkpoint Inhibitors	10.3390/biom10071061	Review	2020	Not an original study
Epigenetic modifications: Critical participants of the PD-L1 regulatory mechanism in solid tumors (Review)	10.3892/ijo.2022.5424	Review	2022	Not an original study
Epigenetic regulation of immune checkpoints: another target for cancer immunotherapy?	10.2217/imt-2016-0111	Review	2017	Not an original study
Epigenetic regulators of programmed death-ligand 1 expression in human cancers	10.1016/j.trsl.2018.05.011	Review	2018	Not an original study
Epigenetics in Health and Disease	10.1007/978-981-15-3449-2_1	Editorial Material; Boc	2020	Not an original study
Epigenomic alterations in cancer: mechanisms and therapeutic potential	10.1042/CS20210449	Review	2022	Not an original study
Epitranscriptomic Approach: To Improve the Efficacy of ICB Therapy by Co-Targeting Intracellular Checkpoint CISH	10.3390/cells10092250	Review	2021	Not an original study
Epstein-Barr virus-encoded miR-BART5-5p upregulates PD-L1 through PIAS3/pSTAT3 modulation, worsening clinical outcomes of PD-L1-positive gastric carcinomas	10.1007/s10120-020-01059-3	Article	2020	Not experimentally demonstrating a direct association between the ICI effect on changes in the levels of miRNAs or vice versa
Epstein–Barr Virus Epithelial Cancers—A Comprehensive Understanding to Drive Novel Therapies	10.3389/fimmu.2021.734293	Review	2021	Not an original study
Essential updates 2017/2018: Recent topics in the treatment and research of gastric cancer in Japan	10.1002/ags3.12284	Review	2019	Not an original study
Evaluate the diagnostic and prognostic value of NUSAP1 in papillary thyroid carcinoma and identify the relationship with genes, proteins, and immune factors	10.1186/s12957-022-02652-9	Article	2022	Not experimentally demonstrating a direct association between the ICI effect on changes in the levels of miRNAs or vice versa
Evaluating prognostic value and relevant gene signatures of tumor microenvironment characterization in esophageal carcinoma	10.21037/jgo-21-371	Article	2021	Not experimentally demonstrating a direct association between the ICI effect on changes in the levels of miRNAs or vice versa
Evaluation of circular RNA profiling in metastatic melanoma patients treated with immune checkpoint blockade		Conference Abstract	2020	Not an original study
Evolving Role of Oncolytic Virotherapy: Challenges and Prospects in Clinical Practice		Review	2021	Not an original study
Exosomal circCMTM3 promotes the pathogenesis of hepatocellular carcinoma via angiogenesis	10.1111/hepr.13719	Editorial	2021	Not an original study
Exosomal miRNA-146a is downregulated in clear cell renal cell carcinoma patients with severe immune-related adverse events	10.1016/j.ncrna.2022.06.004	Article	2022	Not experimentally demonstrating a direct association between the ICI effect on changes in the levels of miRNAs or vice versa
Exosome miR-27a-3p secreted from adipocytes targets ICOS to promote antitumor immunity in lung adenocarcinoma	10.1111/1759-7714.13411	Article	2020	Not experimentally demonstrating a direct association between the ICI effect on changes in the levels of miRNAs or vice versa
Exosome-derived miR-142-5p remodels lymphatic vessels and induces IDO to promote immune privilege in the tumour microenvironment	10.1038/s41418-020-00618-6	Article	2021	Not experimentally demonstrating a direct association between the ICI effect on changes in the levels of miRNAs or vice versa
Exosomes carrying immune checkpoints, a promising therapeutic approach in cancer treatment	10.1007/s12032-022-01781-1	Review	2022	Not an original study
Exploration of Redox-Related Molecular Patterns and the Redox Score for Prostate Cancer	10.1155/2021/4548594	Article	2021	Not focused on the miRNA/ICI relationship
Exploring microsatellite instability in patients with advanced hepatocellular carcinoma and its tumor microenvironment	10.1002/jgh3.12660	Article	2021	Not focused on ICIs
Expression of PD-L1 in EBV-associated malignancies	10.1016/j.intimp.2021.107553	Review	2021	Not an original study
Expression patterns of platinum resistance-related genes in lung adenocarcinoma and related clinical value models	10.3389/fgene.2022.993322	Article	2022	Not focused on the miRNA/ICI relationship
Extracellular Vesicles as Mediators of Therapy Resistance in the Breast Cancer Microenvironment	10.3390/biom12010132	Review	2022	Not an original study
Extracellular vesicles in tumor diagnosis: A mini-review	10.2174/1573405616666201209103154	Short Survey	2021	Not an original study
Extracellular vesicles released by CD40/IL-4-stimulated CLL cells confer altered functional properties to CD4(+) T cells		Article	2016	Not experimentally demonstrating a direct association between the ICI effect on changes in the levels of miRNAs or vice versa
Extranodal natural killer/T-cell lymphoma, nasal type: Basic science and clinical progress	10.3389/fped.2019.00141	Review	2019	Not an original study
Fascin in gynecological cancers: An update of the literature	10.3390/cancers13225760	Review	2021	Not an original study
Ferroptosis: A New Promising Target for Ovarian Cancer Therapy	10.7150/ijms.76480	Review	2022	Not an original study
Fibroblast-derived LPP as a biomarker for treatment response and therapeutic target in gastric cancer	10.1016/j.omto.2022.01.008	Article	2022	Not experimentally demonstrating a direct association between the ICI effect on changes in the levels of miRNAs or vice versa
First-line immunotherapy or angiogenesis inhibitor plus chemotherapy for HER2-altered NSCLC: a retrospective real-world POLISH study	10.1177/17588359221082339	Article	2022	Not focused on miRNAs
Focus on PD-1/PD-L1 as a Therapeutic Target in Ovarian Cancer	10.3390/ijms232012067	Review	2022	Not an original study
From COPD to Lung Cancer: Mechanisms Linking, Diagnosis, Treatment, and Prognosis	10.2147/COPD.S380732	Review	2022	Not an original study
From Liver Cirrhosis to Cancer: The Role of Micro-RNAs in Hepatocarcinogenesis	10.3390/ijms22031492	Review	2021	Not an original study
Fueling immune checkpoint blockade with oncolytic viruses: Current paradigms and challenges ahead	10.1016/j.canlet.2022.215937	Review	2022	Not an original study
Futuristic approach to cancer treatment	10.1016/j.gene.2021.145906	Review	2021	Not an original study
Gender specific eRNA TBX5-AS1 as the immunological biomarker for male patients with lung squamous cell carcinoma in pan-cancer screening	10.7717/peerj.12536	Article	2021	Not experimentally demonstrating a direct association between the ICI effect on changes in the levels of miRNAs or vice versa
Gene signatures with therapeutic value: emerging perspective for personalized immunotherapy in renal cancer	10.2217/imt-2021-0187	Review	2021	Not an original study
Generation, secretion and degradation of cancer immunotherapy target PD-L1	10.1007/s00018-022-04431-x	Review	2022	Not an original study
Genetic and clinical characterization of B7-H3 (CD276) expression and epigenetic regulation in diffuse brain glioma	10.1111/cas.13744	Article	2018	Not experimentally demonstrating a direct association between the ICI effect on changes in the levels of miRNAs or vice versa
Genetic and epigenetic strategies for advancing ovarian cancer immunotherapy	10.1080/14712598.2019.1602605	Review	2019	Not an original study
Genome Instability and Long Noncoding RNA Reveal Biomarkers for Immunotherapy and Prognosis and Novel Competing Endogenous RNA Mechanism in Colon Adenocarcinoma	10.3389/fcell.2021.740455	Article	2021	Not experimentally demonstrating a direct association between the ICI effect on changes in the levels of miRNAs or vice versa
Genomic characterization of small cell bladder cancer		Conference Abstract	2020	Not an original study
Genomic, Pathway Network, and Immunologic Features Distinguishing Squamous Carcinomas	10.1016/j.celrep.2018.03.063	Article	2018	Not focused on the miRNA/ICI relationship
GINS4 might be a novel prognostic immune-related biomarker of not only esophageal squamous cell carcinoma and other cancers	10.1186/s12920-022-01223-x	Article	2022	Not experimentally demonstrating a direct association between the ICI effect on changes in the levels of miRNAs or vice versa
Glycolysis-Related LINC02432/Hsa-miR-98-5p/HK2 Axis Inhibits Ferroptosis and Predicts Immune Infiltration, Tumor Mutation Burden, and Drug Sensitivity in Pancreatic Adenocarcinoma	10.3389/fphar.2022.937413	Article	2022	Not experimentally demonstrating a direct association between the ICI effect on changes in the levels of miRNAs or vice versa
Gut microbiota: a new player in regulating immune- and chemo-therapy efficacy	10.20517/cdr.2020.04	Review	2020	Not an original study
Gynocare update: Modern strategies to improve diagnosis and treatment of rare gynecologic tumors—current challenges and future directions	10.3390/cancers13030493	Review	2021	Not an original study
Harnessing the epigenome to boost immunotherapy response in non-small cell lung cancer patients	10.1177/17588359211006947	Review	2021	Not an original study
Harnessing the therapeutic potential of extracellular vesicles for cancer treatment	10.1016/j.semcancer.2021.05.001	Review	2021	Not an original study
Harnessing tumorous flaws for immune supremacy: is miRNA-155 the weak link in breast cancer progression?	10.1172/JCI163010	Review	2022	Not an original study
HCG18 Participates in Vascular Invasion of Hepatocellular Carcinoma by Regulating Macrophages and Tumor Stem Cells	10.3389/fcell.2021.707073	Article	2021	Not experimentally demonstrating a direct association between the ICI effect on changes in the levels of miRNAs or vice versa
Hepatocellular carcinoma: An overview of the changing landscape of treatment options	10.2147/JHC.S300182	Review	2021	Not an original study
Hereditary diffuse gastric cancer therapeutic roadmap: current and novel approaches in a nutshell	10.1177/1758835920967238	Review	2020	Not an original study
HIF1α epigenetically repressed macrophages via CRISPR/Cas9-EZH2 system for enhanced cancer immunotherapy	10.1016/j.bioactmat.2021.02.008	Article	2021	Not experimentally demonstrating a direct association between the ICI effect on changes in the levels of miRNAs or vice versa
High dose androgen suppresses natural killer cytotoxicity of castration-resistant prostate cancer cells via altering AR/circFKBP5/miRNA-513a-5p/PD-L1 signals	10.1038/s41419-022-04956-w	Article	2022	Not experimentally demonstrating a direct association between the ICI effect on changes in the levels of miRNAs or vice versa
HLA-G: Too Much or Too Little? Role in Cancer and Autoimmune Disease	10.3389/fimmu.2022.796054	Review	2022	Not an original study
hsa_circ_0003222 accelerates stemness and progression of non-small cell lung cancer by sponging miR-527	10.1038/s41419-021-04095-8	Article	2021	Not experimentally demonstrating a direct association between the ICI effect on changes in the levels of miRNAs or vice versa
Hsa-miR-9-5p-Mediated TSPAN9 Downregulation Is Positively Related to Both Poor Hepatocellular Carcinoma Prognosis and the Tumor Immune Infiltration	10.1155/2022/9051229	Article	2022	Not experimentally demonstrating a direct association between the ICI effect on changes in the levels of miRNAs or vice versa
Human endogenous retrovirus-H long terminal repeat-associating 2: The next immune checkpoint for antitumour therapy	10.1016/j.ebiom.2022.103987	Review	2022	Not an original study
Hypoxia-Related lncRNA Correlates With Prognosis and Immune Microenvironment in Lower-Grade Glioma	10.3389/fimmu.2021.731048	Article	2021	Not experimentally demonstrating a direct association between the ICI effect on changes in the levels of miRNAs or vice versa
Identification and characterization of novel CD274 (PD-L1) regulating microRNAs and their functional relevance in melanoma	10.1002/ctm2.934	Article	2022	Article included in qualitative synthesis
Identification and functional characterization of the miRNA-gene regulatory network in chronic myeloid leukemia lineage negative cells	10.1038/srep32493	Article	2016	Not focused on ICIs
Identification and validation of a miRNA-related expression signature for tumor mutational burden in colorectal cancer	10.1186/s12957-021-02137-1	Article	2021	Not experimentally demonstrating a direct association between the ICI effect on changes in the levels of miRNAs or vice versa
Identification and Validation of an Immune-Related lncRNA Signature to Facilitate Survival Prediction in Gastric Cancer	10.3389/fonc.2021.666064	Article	2021	Not experimentally demonstrating a direct association between the ICI effect on changes in the levels of miRNAs or vice versa
Identification and Validation of Long Non-Coding RNA LCIIAR as a Biomarker in LUAD	10.3389/fonc.2022.933071	Article	2022	Not experimentally demonstrating a direct association between the ICI effect on changes in the levels of miRNAs or vice versa
Identification of a competing endogenous RNA network related to immune signature in clear cell renal cell carcinoma		Article	2021	Not experimentally demonstrating a direct association between the ICI effect on changes in the levels of miRNAs or vice versa
Identification of a Ferroptosis-Related Long Noncoding RNA Prognostic Signature and Its Predictive Ability to Immunotherapy in Hepatocellular Carcinoma	10.3389/fgene.2021.682082	Article	2021	Not focused on miRNAs
Identification of a miRNA-based non-invasive predictive biomarker of response to target therapy in BRAF-mutant melanoma	10.7150/thno.77761	Article	2022	Not experimentally demonstrating a direct association between the ICI effect on changes in the levels of miRNAs or vice versa
Identification of a novel six autophagy-related genes signature for the prognostic and a miRNA-related autophagy predictor for anti-PD-1 therapy responses in prostate cancer	10.1186/s12885-020-07725-0	Article	2021	Not experimentally demonstrating a direct association between the ICI effect on changes in the levels of miRNAs or vice versa
Identification of a pyroptosis-related lncRNA risk model for predicting prognosis and immune response in colon adenocarcinoma	10.1186/s12957-022-02572-8	Article	2022	Not experimentally demonstrating a direct association between the ICI effect on changes in the levels of miRNAs or vice versa
Identification of Autophagy Related circRNA-miRNA-mRNA-Subtypes Network With Radiotherapy Responses and Tumor Immune Microenvironment in Non-small Cell Lung Cancer	10.3389/fgene.2021.730003	Article	2021	Not experimentally demonstrating a direct association between the ICI effect on changes in the levels of miRNAs or vice versa
Identification of CTLA-4 associated with tumor microenvironment and competing interactions in triple negative breast cancer by co-expression network analysis	10.7150/jca.46301	Article	2020	Not experimentally demonstrating a direct association between the ICI effect on changes in the levels of miRNAs or vice versa
Identification of immune checkpoint inhibitors and biomarkers among STAT family in stomach adenocarcinoma		Article	2020	Not experimentally demonstrating a direct association between the ICI effect on changes in the levels of miRNAs or vice versa
Identification of Immune Modulatory miRNAs by miRNA Enrichment via RNA Affinity Purification		Article	2019	Not experimentally demonstrating a direct association between the ICI effect on changes in the levels of miRNAs or vice versa
Identification of Immune-Related Genes for Establishment of Prognostic Index in Hepatocellular Carcinoma	10.3389/fcell.2021.760079	Article	2021	Not experimentally demonstrating a direct association between the ICI effect on changes in the levels of miRNAs or vice versa
Identification of m6A modification patterns and development of m6A–hypoxia prognostic signature to characterize tumor microenvironment in triple-negative breast cancer	10.3389/fimmu.2022.978092	Article	2022	Not focused on miRNAs
Identification of MicroRNA-mRNA Networks in Melanoma and Their Association with PD-1 Checkpoint Blockade Outcomes	10.3390/cancers13215301	Article	2021	Not experimentally demonstrating a direct association between the ICI effect on changes in the levels of miRNAs or vice versa
Identification of microRNAs that target PD-L1 in mismatch repair-deficient colorectal cancer		Conference Abstract	2018	Not an original study
Identification of Novel Biomarkers for Predicting Prognosis and Immunotherapy Response in Head and Neck Squamous Cell Carcinoma Based on ceRNA Network and Immune Infiltration Analysis	10.1155/2021/4532438	Article	2021	Not experimentally demonstrating a direct association between the ICI effect on changes in the levels of miRNAs or vice versa
Identification of Novel Prognostic Signatures for Clear Cell Renal Cell Carcinoma Based on ceRNA Network Construction and Immune Infiltration Analysis	10.1155/2022/4033583	Article	2022	Not experimentally demonstrating a direct association between the ICI effect on changes in the levels of miRNAs or vice versa
Identification of serum microRNAs predicting the response of esophageal squamous-cell carcinoma to nivolumab	10.1093/jjco/hyz146	Article	2020	Not experimentally demonstrating a direct association between the ICI effect on changes in the levels of miRNAs or vice versa
Identifies microtubule-binding protein CSPP1 as a novel cancer biomarker associated with ferroptosis and tumor microenvironment		Article	2022	Not experimentally demonstrating a direct association between the ICI effect on changes in the levels of miRNAs or vice versa
Identifying tumor immunity-associated molecular features in liver hepatocellular carcinoma by multi-omics analysis	10.3389/fmolb.2022.960457	Article	2022	Not experimentally demonstrating a direct association between the ICI effect on changes in the levels of miRNAs or vice versa
IFN-I-tolerant oncolytic Semliki Forest virus in combination with anti-PD1 enhances T cell response against mouse glioma	10.1016/j.omto.2021.03.008	Article	2021	Not focused on miRNAs
Immune checkpoint inhibitor induces cardiac injury through polarizing macrophages via modulating microRNA-34a/Kruppel-like factor 4 signaling	10.1038/s41419-020-02778-2	Article	2020	Article included in qualitative synthesis
Immune checkpoint inhibitors as mediators for immunosuppression by cancer-associated fibroblasts: A comprehensive review	10.3389/fimmu.2022.996145	Review	2022	Not an original study
Immune checkpoint inhibitors for genitourinary cancers: Treatment indications, investigational approaches and biomarkers	10.3390/cancers13215415	Review	2021	Not an original study
Immune checkpoint inhibitors in HCC: Cellular, molecular and systemic data	10.1016/j.semcancer.2022.01.005	Review	2022	Not an original study
Immune checkpoints in osteosarcoma: Recent advances and therapeutic potential	10.1016/j.canlet.2022.215887	Review	2022	Not an original study
Immune Modulatory Short Noncoding RNAs Targeting the Glioblastoma Microenvironment	10.3389/fonc.2021.682129	Review	2021	Not an original study
Immune Prophets of Lung Cancer: The Prognostic and Predictive Landscape of Cellular and Molecular Immune Markers	10.1016/j.tranon.2018.04.006	Review	2018	Not an original study
Immunogenomic correlates of immune-related adverse events for anti–programmed cell death 1 therapy	10.3389/fimmu.2022.1032221	Article	2022	Not focused on miRNAs
Immunologic and tumor responses of pegilodecakin with 5-FU/LV and oxaliplatin (FOLFOX) in pancreatic ductal adenocarcinoma (PDAC)	10.1007/s10637-020-01000-6	Article	2021	Not focused on miRNAs
Immunology Meets Bioengineering: Improving the Effectiveness of Glioblastoma Immunotherapy	10.3390/cancers14153698	Review	2022	Not an original study
Immunomodulatory role for MicroRNAs: Regulation of PD-1/PD-L1 and CTLA-4 immune checkpoints expression	10.1016/j.gene.2020.144888	Review	2020	Not an original study
Immunotherapy and Gene Therapy for Oncoviruses Infections: A Review	10.3390/v13050822	Review	2021	Not an original study
Immunotherapy of cholangiocarcinoma: Therapeutic strategies and predictive biomarkers	10.1016/j.canlet.2022.215853	Review	2022	Not an original study
Improvement in survival end points of patients with metastatic renal cell carcinoma through sequential targeted therapy	10.1016/j.ctrv.2016.09.002	Review	2016	Not an original study
Improving PARP inhibitor efficacy in high-grade serous ovarian carcinoma: A focus on the immune system	10.3389/fgene.2022.886170	Review	2022	Not an original study
Inflammatory microenvironment in the initiation and progression of bladder cancer	10.18632/oncotarget.21565	Review	2017	Not an original study
Inflation of tumor mutation burden by tumor-only sequencing in under-represented groups	10.1038/s41698-021-00164-5	Article	2021	Not focused on miRNAs
Inhibition of arginase by CB-1158 blocks myeloid cell-mediated immune suppression in the tumor microenvironment		Article	2017	Not focused on miRNAs
Inhibition of CXCR4 by MicroRNA-1192 Reduces the Activation of Th17 Cells and Expression of Inflammation Factors in a Mouse Model of Vulvovaginal Candidiasis	10.1159/000494475	Article	2018	Not focused on cancer
Inhibition of PARP Potentiates Immune Checkpoint Therapy through miR-513/PD-L1 Pathway in Hepatocellular Carcinoma	10.1155/2022/6988923	Article	2022	Not experimentally demonstrating a direct association between the ICI effect on changes in the levels of miRNAs or vice versa
Innovative Therapy, Monoclonal Antibodies and Beyond	10.1016/j.cytogfr.2017.10.002	Short Survey	2017	Not an original study
Insights into an immunotherapeutic approach to combat multidrug resistance in hepatocellular carcinoma	10.3390/ph14070656	Review	2021	Not an original study
Insights into molecular therapy of glioma: current challenges and next generation blueprint	10.1038/aps.2016.167	Review	2017	Not an original study
Integrated genomic analysis of recurrence-associated small non-coding RNAs in oesophageal cancer	10.1136/gutjnl-2015-311238	Article	2017	Not experimentally demonstrating a direct association between the ICI effect on changes in the levels of miRNAs or vice versa
Integrated Multi-Omics Analysis Identified PTPRG and CHL1 as Key Regulators of Immunophenotypes in Clear Cell Renal Cell Carcinoma(ccRCC)		Article	2022	Not experimentally demonstrating a direct association between the ICI effect on changes in the levels of miRNAs or vice versa
Integrated transcriptional-phenotypic analysis captures systemic immunomodulation following antiangiogenic therapy in renal cell carcinoma patients	10.1002/ctm2.434	Article	2021	Not experimentally demonstrating a direct association between the ICI effect on changes in the levels of miRNAs or vice versa
Integrative analyses of prognosis, tumor immunity, and ceRNA network of the ferroptosis-associated gene FANCD2 in hepatocellular carcinoma	10.3389/fgene.2022.955225	Article	2022	Not experimentally demonstrating a direct association between the ICI effect on changes in the levels of miRNAs or vice versa
Integrative Analysis of Multi-omics Data Identified EGFR and PTGS2 as Key Nodes in a Gene Regulatory Network Related to Immune Phenotypes in Head and Neck Cancer	10.1158/1078-0432.CCR-19-3997	Article	2020	Not experimentally demonstrating a direct association between the ICI effect on changes in the levels of miRNAs or vice versa
Integrative cancer genomics in the era of precision cancer medicine	10.1038/s10038-021-00970-6	Editorial Material	2021	Not an original study
Interaction of Gut Microbiome and Host microRNAs with the Occurrence of Colorectal and Breast Cancer and Their Impact on Patient Immunity		Review	2021	Not an original study
Interactome analysis of gene expression profiles identifies CDC6 as a potential therapeutic target modified by miR-215-5p in hepatocellular carcinoma	10.7150/ijms.51145	Article	2020	Not experimentally demonstrating a direct association between the ICI effect on changes in the levels of miRNAs or vice versa
Interplay between programmed death-ligand 1 and non-coding RNAs		Review	2022	Not an original study
Interrogating the interplay of angiogenesis and immunity in metastatic colorectal cancer	10.5662/wjm.v12.i1.43	Review	2022	Not an original study
Intrahepatic Cholangiocarcinoma: Genomic Heterogeneity Between Eastern and Western Patients	10.1200/PO.18.00414	Article	2020	Not focused on miRNAs
Intratumoral immunosuppression profiles in 11q-deleted neuroblastomas provide new potential therapeutic targets	10.1002/1878-0261.12868	Article	2021	Not focused on ICIs
Ionizing Radiation-Inducible miR-27b Suppresses Leukemia Proliferation via Targeting Cyclin A2	10.1016/j.ijrobp.2014.04.055	Article	2014	Not focused on ICIs
Is the scavenger receptor MARCO a new immune checkpoint?	10.4161/21624011.2014.955709	Review	2014	Not an original study
Kaposi's Sarcoma-Associated Herpesvirus MicroRNAs Target GADD45B To Protect Infected Cells from Cell Cycle Arrest and Apoptosis	10.1128/JVI.02045-16	Article	2017	Not focused on ICIs
KCNN4 may weaken anti-tumor immune response via raising Tregs and diminishing resting mast cells in clear cell renal cell carcinoma	10.1186/s12935-022-02626-7	Article	2022	Not experimentally demonstrating a direct association between the ICI effect on changes in the levels of miRNAs or vice versa
KDM1A Promotes Immunosuppression in Hepatocellular Carcinoma by Regulating PD-L1 through Demethylating MEF2D	10.1155/2021/9965099	Article	2021	Not experimentally demonstrating a direct association between the ICI effect on changes in the levels of miRNAs or vice versa
Key questions about the checkpoint blockade-are microRNAs an answer?	10.20892/j.issn.2095-3941.2018.0006	Review	2018	Not an original study
Landscape of EBV-positive gastric cancer	10.1007/s10120-021-01215-3	Review	2021	Not an original study
Landscape of extracellular vesicles in the tumour microenvironment: Interactions with stromal cells and with non-cell components, and impacts on metabolic reprogramming, horizontal transfer of neoplastic tra	10.1016/j.semcancer.2021.01.007	Review	2021	Not an original study
Large-Scale Profiling of Extracellular Vesicles Identified miR-625-5p as a Novel Biomarker of Immunotherapy Response in Advanced Non-Small-Cell Lung Cancer Patients	10.3390/cancers14102435	Article	2022	Not experimentally demonstrating a direct association between the ICI effect on changes in the levels of miRNAs or vice versa
Lessons From Immune Checkpoint Inhibitor Trials in Hepatocellular Carcinoma	10.3389/fimmu.2021.652172	Review	2021	Not an original study
Leukemia cell-derived microvesicles induce T cell exhaustion via miRNA delivery	10.1080/2162402X.2018.1448330	Article	2018	Not experimentally demonstrating a direct association between the ICI effect on changes in the levels of miRNAs or vice versa
Life and Death Decision-Making by p53 and Implications for Cancer Immunotherapy	10.1016/j.trecan.2020.10.005	Review	2021	Not an original study
LILRB4 promotes tumor metastasis by regulating MDSCs and inhibiting miR-1 family miRNAs	10.1080/2162402X.2022.2060907	Article	2022	Not experimentally demonstrating a direct association between the ICI effect on changes in the levels of miRNAs or vice versa
LincRNA-p21 promotes p21-mediated cell cycle arrest in benzene-induced hematotoxicity by sponging miRNA-17-5p*	10.1016/j.envpol.2021.118706	Article	2022	Not focused on ICIs
Liquid biopsies in head and neck cancer: Current state and future challenges	10.3390/cancers13081874	Review	2021	Not an original study
Liquid Biopsies to Evaluate Immunogenicity of Gynecological/Breast Tumors: On the Way to Blood-Based Biomarkers for Immunotherapies	10.1159/000510509	Review	2020	Not an original study
Liquid biopsy-based biosensors for mrd detection and treatment monitoring in non-small cell lung cancer (Nsclc)	10.3390/bios11100394	Review	2021	Not an original study
LncRNA HEIH/miR-939-5p interplay modulates triple-negative breast cancer progression through NOS2-induced nitric oxide production	10.1002/jcp.30234	Article	2021	Not experimentally demonstrating a direct association between the ICI effect on changes in the levels of miRNAs or vice versa
lncRNA-mediated ceRNA network in bladder cancer		Review	2022	Not an original study
Long non-coding RNAs and microRNAs as crucial regulators in cardio-oncology	10.1186/s13578-022-00757-y	Review	2022	Not an original study
Long Non-Coding RNAs as Epigenetic Regulators of Immune Checkpoints in Cancer Immunity	10.3390/cancers15010184	Review	2022	Not an original study

Long non-coding RNAs XIST and MALAT1 hijack the PD-L1 regulatory signaling pathway in breast cancer subtypes	10.3892/ol.2021.12854	Article	2021	Not experimentally demonstrating a direct association between the ICI effect on changes in the levels of miRNAs or vice versa
Long Noncoding RNA TUG1 Inhibits Tumor Progression through Regulating Siglec-15-Related Anti-Immune Activity in Hepatocellular Carcinoma	10.1155/2022/9557859	Article	2022	Not experimentally demonstrating a direct association between the ICI effect on changes in the levels of miRNAs or vice versa
Longer-term follow-up of a phase 2 study (checkmate 063) of nivolumab in patients with advanced, refractory squamous non-small cell lung cancer		Conference Abstract	2015	Not an original study
Low-dose radiation treatment enhances systemic antitumor immune responses by overcoming the inhibitory stroma	10.1136/jitc-2020-000537	Article	2020	Not focused neither on miRNA nor on ICIs
Lymphatic vessels, miRNAs, and CAR T cells in tumor immunology	10.1631/jzus.B1910003	Review	2020	Not an original study
Machine learning and bioinformatics analysis revealed classification and potential treatment strategy in stage 3–4 NSCLC patients	10.1186/s12920-022-01184-1	Article	2022	Not experimentally demonstrating a direct association between the ICI effect on changes in the levels of miRNAs or vice versa
Machine learning-based exceptional response prediction of nivolumab monotherapy with circulating microRNAs in non-small cell lung cancer	10.1016/j.lungcan.2022.09.004	Article	2022	Not experimentally demonstrating a direct association between the ICI effect on changes in the levels of miRNAs or vice versa
Macrophage reprogramming for anticancer therapy		Conference Abstract	2016	Not an original study
Macrophage targeting in cancer	10.1111/nyas.14377	Review	2021	Not an original study
Macrophages as tools and targets in cancer therapy	10.1038/s41573-022-00520-5	Review	2022	Not an original study
Malignant Pleural Mesothelioma: State-of-the-Art on Current Therapies and Promises for the Future	10.3389/fonc.2019.01519	Review	2020	Not an original study
Manipulating immune system using nanoparticles for an effective cancer treatment: Combination of targeted therapy and checkpoint blockage miRNA	10.1016/j.jconrel.2020.09.034	Article	2021	Not experimentally demonstrating a direct association between the ICI effect on changes in the levels of miRNAs or vice versa
Markers of Aggressiveness in Pituitary Tumors: Update and Perspectives	10.3390/jcm11216508	Editorial	2022	Not an original study
Mathematical Modeling of the Metastatic Colorectal Cancer Microenvironment Defines the Importance of Cytotoxic Lymphocyte Infiltration and Presence of PD-L1 on Antigen Presenting Cells		Article	2020	Not focused on miRNAs
Mechanisms of MHC-I Downregulation and Role in Immunotherapy Response	10.3389/fimmu.2022.844866	Review	2022	Not an original study
Mechanisms of tumor-associated myeloid cells in modulating host immune microenvironment and metastatic progression	10.1158/1538-7445.SABCS18-4500	Conference Abstract	2019	Not an original study
Melanoma genomics: a state-of-the-art review of practical clinical applications*	10.1111/bjd.20421	Review	2021	Not an original study
Micro-RNAs, the Cornerstones of the Future of Radiobiology in Head and Neck Cancers?	10.3390/curroncol29020069	Review	2022	Not an original study
Microenvironment Remodeling and Subsequent Clinical Implications in Diffuse Large B-Cell Histologic Variant of Richter Syndrome	10.3389/fimmu.2020.594841	Review	2020	Not an original study
Microenvironmental oxygen pressure orchestrates an anti- and pro-tumoral γδ T cell equilibrium via tumor-derived exosomes	10.1038/s41388-018-0627-z	Article	2019	Article included in qualitative synthesis
microRNA 125a Regulates MHC-I Expression on Esophageal Adenocarcinoma Cells, Associated With Suppression of Antitumor Immune Response and Poor Outcomes of Patients		Article	2018	Not experimentally demonstrating a direct association between the ICI effect on changes in the levels of miRNAs or vice versa
MicroRNA based theranostics for brain cancer: basic principles	10.1186/s13046-019-1180-5	Review	2019	Not an original study
microRNA signature of T cell exhaustion	10.1158/1538-7445.AM2018-5390	Meeting Abstract	2018	Not an original study
MicroRNA-139 is a predictor of prostate cancer recurrence and inhibits growth and migration of prostate cancer cells through cell cycle arrest and targeting IGF1R and AXL	10.1002/pros.23871	Article	2019	Not focused on ICIs
MicroRNA-140 suppresses osteosarcoma tumor growth by enhancing anti-tumor immune response and blocking mTOR signaling		Article	2018	Not experimentally demonstrating a direct association between the ICI effect on changes in the levels of miRNAs or vice versa
MicroRNA-146a regulates immune-related adverse events caused by immune checkpoint inhibitors	10.1172/jci.insight.132334	Article	2020	Article included in qualitative synthesis
MicroRNA-155 induction via TNF-α and IFN-γ suppresses expression of programmed death ligand-1 (PD-L1) in human primary cells	10.1074/jbc.M117.809053	Article	2017	Not experimentally demonstrating a direct association between the ICI effect on changes in the levels of miRNAs or vice versa
MicroRNA-199a-5p functions as a tumor suppressor in oral squamous cell carcinoma via targeting the IKK beta/NF-kappa B signaling pathway	10.3892/ijmm.2019.4083	Article	2019	Not focused on ICIs
MicroRNA-200b is a potential biomarker of the expression of PD-L1 in patients with lung cancer	10.1111/1759-7714.13653	Article	2020	Not experimentally demonstrating a direct association between the ICI effect on changes in the levels of miRNAs or vice versa
microRNA-20b-5p overexpression combing Pembrolizumab potentiates cancer cells to radiation therapy via repressing programmed death-ligand 1	10.1080/21655979.2021.2014617	Article	2022	Article included in qualitative synthesis
MicroRNA-224 negatively regulates p21 expression during late neoplastic progression in inflammatory bowel disease	10.1097/MIB.0b013e31827e78eb	Article	2013	Not experimentally demonstrating a direct association between the ICI effect on changes in the levels of miRNAs or vice versa
MicroRNA-375 Inhibits Growth and Enhances Radiosensitivity in Oral Squamous Cell Carcinoma by Targeting Insulin Like Growth Factor 1 Receptor	10.1159/000479913	Article	2017	Not experimentally demonstrating a direct association between the ICI effect on changes in the levels of miRNAs or vice versa
MicroRNA-497-5p down-regulation increases PD-L1 expression in clear cell renal cell carcinoma	10.1080/1061186X.2018.1479755	Article	2019	Not experimentally demonstrating a direct association between the ICI effect on changes in the levels of miRNAs or vice versa
MicroRNA-519d-3p antagonizes osteosarcoma resistance against cisplatin by targeting PD-L1	10.1002/mc.23370	Article	2022	Not experimentally demonstrating a direct association between the ICI effect on changes in the levels of miRNAs or vice versa
MicroRNA-regulated transcriptome analysis identifies four major subtypes with prognostic and therapeutic implications in prostate cancer		Article	2021	Not experimentally demonstrating a direct association between the ICI effect on changes in the levels of miRNAs or vice versa
MicroRNA-140 suppresses Helicobacter pylori-positive gastric cancer growth by enhancing the antitumor immune response		Article	2019	Not experimentally demonstrating a direct association between the ICI effect on changes in the levels of miRNAs or vice versa
MicroRNAs aid the assessment of programmed death ligand 1 expression in patients with non-small cell lung cancer	10.3892/ol.2019.10207	Article	2019	Not experimentally demonstrating a direct association between the ICI effect on changes in the levels of miRNAs or vice versa
MicroRNAs are implicated in the suppression of CD4(+)CD25(-) conventional T cell proliferation by CD4(+)CD25(+) regulatory T cells	10.1016/j.molimm.2014.10.001	Article	2015	Not experimentally demonstrating a direct association between the ICI effect on changes in the levels of miRNAs or vice versa
MicroRNAs as Key Players in Melanoma Cell Resistance to MAPK and Immune Checkpoint Inhibitors	10.3390/ijms21124544	Review	2020	Not an original study
MicroRNAs as Predictive Biomarkers of Resistance to Targeted Therapies in Gastrointestinal Tumors	10.3390/biomedicines9030318	Review	2021	Not an original study
MicroRNAs at the Crossroad between Immunoediting and Oncogenic Drivers in Hepatocellular Carcinoma	10.3390/biom12070930	Review	2022	Not an original study
MicroRNAs Change the Landscape of Cancer Resistance		Review	2018	Not an original study
MicroRNAs Expression Patterns Predict Tumor Mutational Burden in Colorectal Cancer	10.3389/fonc.2020.550986	Article	2021	Not experimentally demonstrating a direct association between the ICI effect on changes in the levels of miRNAs or vice versa
MicroRNAs in the tumor microenvironment	10.1007/978-3-030-50224-9_1	Chapter	2020	Not an original study
microRNAs Shape Myeloid Cell-Mediated Resistance to Cancer Immunotherapy	10.3389/fimmu.2020.01214	Review	2020	Not an original study
MicroRNAs that predict the effectiveness of anti-PD-1 therapies in patients with advanced melanoma	10.1016/j.jdermsci.2019.11.010	Article	2020	Not experimentally demonstrating a direct association between the ICI effect on changes in the levels of miRNAs or vice versa
Microsatellite Instability in Colorectal Cancers: Carcinogenesis, Neo-Antigens, Immuno-Resistance and Emerging Therapies	10.3390/cancers13123063	Review	2021	Not an original study
miR-101, miR-548b, miR-554, and miR-1202 are reliable prognosis predictors of the miRNAs associated with cancer immunity in primary central nervous system lymphoma		Article	2020	Not experimentally demonstrating a direct association between the ICI effect on changes in the levels of miRNAs or vice versa
MiR-138 exerts anti-glioma efficacy by targeting immune checkpoints	10.1093/neuonc/nov292	Article	2016	Not experimentally demonstrating a direct association between the ICI effect on changes in the levels of miRNAs or vice versa
MiR-146b-5p regulates IL-23 receptor complex expression in chronic lymphocytic leukemia cells	10.1182/bloodadvances.2021005726	Article	2022	Not experimentally demonstrating a direct association between the ICI effect on changes in the levels of miRNAs or vice versa
miR-148a-3p silences the CANX/MHC-I pathway and impairs CD8(+) T cell-mediated immune attack in colorectal cancer	10.1096/fj.202100235R	Article	2021	Not experimentally demonstrating a direct association between the ICI effect on changes in the levels of miRNAs or vice versa
miR-149-3p reverses CD8(+) T-cell exhaustion by reducing inhibitory receptors and promoting cytokine secretion in breast cancer cells	10.1098/rsob.190061	Article	2019	Not experimentally demonstrating a direct association between the ICI effect on changes in the levels of miRNAs or vice versa
miR-153 suppresses IDO1 expression and enhances CAR T cell immunotherapy	10.1186/s13045-018-0600-x	Article	2018	Not focused on ICIs
miR-18a Mediates Immune Evasion in ER-Positive Breast Cancer through Wnt Signaling	10.3390/cells11101672	Article	2022	Not experimentally demonstrating a direct association between the ICI effect on changes in the levels of miRNAs or vice versa
MiR-200b and miR-155 as predictive biomarkers for the efficacy of chemoradiation in locally advanced head and neck squamous cell carcinoma	10.1016/j.ejca.2017.02.018	Article	2017	Not focused on ICIs
MiR-200c-3p Contrasts PD-L1 Induction by Combinatorial Therapies and Slows Proliferation of Epithelial Ovarian Cancer through Downregulation of β-Catenin and c-Myc	10.3390/cells10030519	Article	2021	Not focused on ICIs
miR-21 depletion in macrophages promotes tumoricidal polarization and enhances PD-1 immunotherapy		Article	2018	Article included in qualitative synthesis
MiR-29c downregulates tumor-expressed B7-H3 to mediate the antitumor NK-cell functions in ovarian cancer		Article	2021	Not experimentally demonstrating a direct association between the ICI effect on changes in the levels of miRNAs or vice versa
Mir-34a as predictor of immunotherapy efficacy in NSCLC patients	10.1200/JCO.2021.39.15_suppl.e21191	Conference Abstract	2021	Not an original study
miR-424(322) reverses chemoresistance via T-cell immune response activation by blocking the PD-L1 immune checkpoint		Article	2016	Not experimentally demonstrating a direct association between the ICI effect on changes in the levels of miRNAs or vice versa
miR-4759 suppresses breast cancer through immune checkpoint blockade	10.1016/j.csbj.2021.12.020	Article	2021	Article included in qualitative synthesis
miR-582 Suppresses the Proliferation of B-Cell Precursor Acute Lymphoblastic Leukemia (BCP-ALL) Cells and Protects Them From Natural Killer Cell-Mediated Cytotoxicity		Article	2022	Article included in qualitative synthesis
MIR-708 promotes phagocytosis to eradicate T-ALL cells by targeting CD47		Article	2018	Article included in qualitative synthesis
MiR155 sensitized B-lymphoma cells to anti-PD-L1 antibody via PD-1/PD-L1-mediated lymphoma cell interaction with CD8+T cells	10.1186/s12943-019-0977-3	Article	2019	Article included in qualitative synthesis
MIR155HG is a prognostic biomarker and associated with immune infiltration and immune checkpoint molecules expression in multiple cancers	10.1002/cam4.2583	Article	2019	Not focused on miRNAs
miR3609 sensitizes breast cancer cells to adriamycin by blocking the programmed death-ligand 1 immune checkpoint	10.1016/j.yexcr.2019.03.025	Article	2019	Not experimentally demonstrating a direct association between the ICI effect on changes in the levels of miRNAs or vice versa
MIR548P and TRAV39 Are Potential Indicators of Tumor Microenvironment and Novel Prognostic Biomarkers of Esophageal Squamous Cell Carcinoma	10.1155/2022/3152114	Article	2022	Not focused on miRNAs
miRNA as a Modulator of Immunotherapy and Immune Response in Melanoma	10.3390/biom11111648	Review	2021	Not an original study
MiRNA based tumor mutation burden diagnostic and prognostic prediction models for endometrial cancer	10.1080/21655979.2021.1947940	Article	2021	Not experimentally demonstrating a direct association between the ICI effect on changes in the levels of miRNAs or vice versa
MiRNA expression patterns are associated with tumor mutational burden in lung adenocarcinoma	10.1080/2162402X.2019.1629260	Article	2019	Not experimentally demonstrating a direct association between the ICI effect on changes in the levels of miRNAs or vice versa
MiRNA set dysregulation in breast cancer cells can contribute to both primary and acquired resistance to immune checkpoint blockade	10.1016/j.ejca.2018.01.033	Meeting Abstract	2018	Not an original study
miRNA- and cytokine-associated extracellular vesicles mediate squamous cell carcinomas	10.1080/20013078.2020.1790159	Article	2020	Not experimentally demonstrating a direct association between the ICI effect on changes in the levels of miRNAs or vice versa
miRNA-506-3p Directly Regulates rs10754339 (A/G) in the Immune Checkpoint Protein B7-H4 in Breast Cancer	10.2174/2211536609666201209152949	Article	2020	Full text unavalaible
miRNA-5119 regulates immune checkpoints in dendritic cells to enhance breast cancer immunotherapy		Article	2020	Not experimentally demonstrating a direct association between the ICI effect on changes in the levels of miRNAs or vice versa
miRNA-Based Feature Classifier Is Associated with Tumor Mutational Burden in Head and Neck Squamous Cell Carcinoma	10.1155/2020/1686480	Article	2020	Not experimentally demonstrating a direct association between the ICI effect on changes in the levels of miRNAs or vice versa
MiRNA-based model for predicting the TMB level in colon adenocarcinoma based on a LASSO logistic regression method	10.1097/MD.0000000000026068	Article	2021	Not experimentally demonstrating a direct association between the ICI effect on changes in the levels of miRNAs or vice versa
miRNA-Based Signature Associated With Tumor Mutational Burden in Colon Adenocarcinoma	10.3389/fonc.2021.634841	Article	2021	Not experimentally demonstrating a direct association between the ICI effect on changes in the levels of miRNAs or vice versa
miRNA-Based Therapeutics in the Era of Immune-Checkpoint Inhibitors	10.3390/ph14020089	Review	2021	Not an original study
miRNA-target Regulatory Network showing interaction between Genes of Immune Checkpoints and Cancer Pathways		Review	2020	Not an original study
miRNAs and lncRNAs as Novel Therapeutic Targets to Improve Cancer Immunotherapy	10.3390/cancers13071587	Review	2021	Not an original study
miRNAs as Key Players in the Management of Cutaneous Melanoma	10.3390/cells9020415	Review	2020	Not an original study
miRNAs in lung cancer. A systematic review identifies predictive and prognostic miRNA candidates for precision medicine in lung cancer	10.1016/j.trsl.2020.11.012	Review	2021	Not an original study
miRNAs in Regulation of Tumor Microenvironment, Chemotherapy Resistance, Immunotherapy Modulation and miRNA Therapeutics in Cancer		Review	2022	Not an original study
Modulating the tumor immune microenvironment with nanoparticles: A sword for improving the efficiency of ovarian cancer immunotherapy	10.3389/fimmu.2022.1057850	Review	2022	Not an original study
Modulation of immunosuppressive cells and noncoding RNAs as immunotherapy in osteosarcoma	10.3389/fimmu.2022.1025532	Short Survey	2022	Not an original study
Molecular and immune targets for Merkel cell carcinoma therapy and prevention	10.1002/mc.23042	Review	2019	Not an original study
Molecular characterization, clinical relevance and immune feature of m7G regulator genes across 33 cancer types	10.3389/fgene.2022.981567	Article	2022	Not focused on miRNAs
Molecular events in the pathogenesis of vulvar squamous cell carcinoma	10.1053/j.semdp.2020.09.010	Review	2021	Not an original study
Molecular landscape of IDH-mutant astrocytoma and oligodendroglioma grade 2 indicate tumor purity as an underlying genomic factor	10.1186/s10020-022-00454-z	Article	2022	Not focused on ICIs
Molecular mechanism for miR-350 in regulating of titanium dioxide nanoparticles in macrophage RAW264.7 cells	10.1016/j.cbi.2017.12.020	Article	2018	Not focused on ICIs
Molecular Mechanisms of Cutaneous Squamous Cell Carcinoma	10.3390/ijms23073478	Review	2022	Not an original study
Molecular mechanisms of pd-1 and pd-l1 activity on a pan-cancer basis: A bioinformatic exploratory study	10.3390/ijms22115478	Article	2021	Not experimentally demonstrating a direct association between the ICI effect on changes in the levels of miRNAs or vice versa
Molecular Mechanisms of Resistance to Immunotherapy and Antiangiogenic Treatments in Clear Cell Renal Cell Carcinoma	10.3390/cancers13235981	Review	2021	Not an original study
Molecular pathology of thymomas: implications for diagnosis and therapy	10.1007/s00428-021-03068-8	Review	2021	Not an original study
Molecular Pathology of Well-Differentiated Gastro-entero-pancreatic Neuroendocrine Tumors	10.1007/s12022-021-09662-5	Review	2021	Not an original study
Molecular Signatures in Hepatocellular Carcinoma: A Step Toward Rationally Designed Cancer Therapy	10.1002/cncr.31257	Review	2018	Not an original study
Msi1 in maintaining breast cancer stem cell involves the AKT/PI3K pathway	10.1158/1538-7445.SABCS15-P1-06-07	Conference Abstract	2016	Not an original study
Multi-layered control of PD-L1 expression in Epstein-Barr virus-associated gastric cancer		Review	2020	Not an original study
Multi-modal molecular programs regulate melanoma cell state	10.1038/s41467-022-31510-1	Article	2022	Not focused on miRNAs
Multicomponent siRNA/miRNA-loaded modified mesoporous silica nanoparticles targeted bladder cancer for a highly effective combination therapy		Article	2022	Not experimentally demonstrating a direct association between the ICI effect on changes in the levels of miRNAs or vice versa
Multidimension Analysis of the Prognostic Value, Immune Regulatory Function, and ceRNA Network of LY6E in Individuals with Colorectal Cancer	10.1155/2022/5164265	Article	2022	Not experimentally demonstrating a direct association between the ICI effect on changes in the levels of miRNAs or vice versa
Multiple endocrine neoplasia type 1 (MEN-1) and neuroendocrine neoplasms (NENs)	10.1016/j.semcancer.2021.04.011	Review	2022	Not an original study
Multiple strategies to improve the therapeutic efficacy of oncolytic herpes simplex virus in the treatment of glioblastoma (Review)	10.3892/ol.2021.12771	Article	2021	Not focused on miRNAs
Myeloid-derived suppressor cells-new and exciting players in lung cancer	10.1186/s13045-020-0843-1	Review	2020	Not an original study
N6-methyladenosine-associated prognostic pseudogenes contribute to predicting immunotherapy benefits and therapeutic agents in head and neck squamous cell carcinoma	10.7150/thno.76689	Article	2022	Not experimentally demonstrating a direct association between the ICI effect on changes in the levels of miRNAs or vice versa
Nano-constructs targeting the primary cellular energy source of cancer cells for modulating tumor progression	10.1016/j.onano.2022.100107	Article	2022	Not focused on the miRNA/ICI relationship
Nanobubble-mediated co-delivery of Ce6 and miR-195 for synergized sonodynamic and checkpoint blockade combination therapy with elicitation of robust immune response in hepatocellular carcinoma	10.1016/j.ejpb.2022.10.017	Article	2022	Not experimentally demonstrating a direct association between the ICI effect on changes in the levels of miRNAs or vice versa
Nanobubbles containing PD-L1 Ab and miR-424 mediated PD-L1 blockade, and its expression inhibition to enable and potentiate hepatocellular carcinoma immunotherapy in mice	10.1016/j.ijpharm.2022.122352	Article	2022	Article included in qualitative synthesis
Nanoparticles for Chemoimmunotherapy Against Triple-Negative Breast Cancer	10.2147/IJN.S388075	Review	2022	Not an original study
Natural Killer Cells as Key Mediators in Type I Diabetes Immunopathology	10.3389/fimmu.2021.722979	Review	2021	Not an original study
Neuroimmune crosstalk and its impact on cancer therapy and research	10.1007/s12672-022-00547-5	Review	2022	Not an original study
Neutrophil: A New Player in Metastatic Cancers	10.3389/fimmu.2020.565165	Review	2020	Not an original study
Next Steps for Immunotherapy in Glioblastoma	10.3390/cancers14164023	Review	2022	Not an original study
Nivolumab alone or in combination with cisplatin plus gemcitabine in Japanese patients with unresectable or recurrent biliary tract cancer: a non-randomised, multicentre, open-label, phase 1 study	10.1016/S2468-1253(19)30086-X	Article	2019	Not focused on miRNAs
Non-Coding RNA in Penile Cancer	10.3389/fonc.2022.812008	Review	2022	Not an original study
Non-coding RNAs: Key players in T cell exhaustion	10.3389/fimmu.2022.959729	Review	2022	Not an original study
Noncoding RNAs as potential mediators of resistance to cancer immunotherapy	10.1016/j.semcancer.2019.11.006	Review	2020	Not an original study
Nonsense-mutation in TP53 improves clinical benefits of PD-1/PD-L1 blockade immunotherapy in advanced urothelial carcinoma through up-regulating PD-L1 expression via miR-34a	10.1016/S0302-2838(21)00833-2	Conference Abstract	2021	Not an original study
Novel biomarkers of gastrointestinal cancer	10.3390/cancers13071501	Editorial	2021	Not an original study
Novel Implications of MicroRNAs, Long Non-coding RNAs and Circular RNAs in Drug Resistance of Esophageal Cancer	10.3389/fcell.2021.764313	Review	2021	Not an original study
Novel insights into the biomarkers and therapies for primary central nervous system lymphoma	10.1177/17588359221093745	Review	2022	Not an original study
Novel macrophage-related gene prognostic index for glioblastoma associated with M2 macrophages and T cell dysfunction	10.3389/fimmu.2022.941556	Article	2022	Not experimentally demonstrating a direct association between the ICI effect on changes in the levels of miRNAs or vice versa
Novel miRNAs as Potential Regulators of PD-1/PD-L1 Immune Checkpoint, and Prognostic Value of MIR9-1 and MIR124-2 Methylation in Ovarian Cancer	10.1134/S0026893320060072	Article	2020	Not experimentally demonstrating a direct association between the ICI effect on changes in the levels of miRNAs or vice versa
Novel possibility for cutaneous melanoma treatment by means of rosmarinic acid action on purinergic signaling	10.1007/s11302-021-09821-7	Review	2022	Not an original study
OncoAlert Round Table Discussions: The Global COVID-19 Experience	10.1200/GO.20.00603	Review	2021	Not an original study
Oncolytic adenovirus: A tool for reversing the tumor microenvironment and promoting cancer treatment (Review)	10.3892/OR.2021.8000	Review	2021	Not an original study
ONCR-177, an Oncolytic HSV-1 Designed to Potently Activate Systemic Antitumor Immunity	10.1158/2326-6066.CIR-20-0609	Article	2021	Not experimentally demonstrating a direct association between the ICI effect on changes in the levels of miRNAs or vice versa
One year in review 2020: Comorbidities, diagnosis and treatment of primary Sjögren's syndrome		Review	2021	Not an original study
Organic/Inorganic Self-Assembled Hybrid Nano-Architectures for Cancer Therapy Applications	10.1002/mabi.202100349	Review	2022	Not an original study
Over-Expression of GUSB Leads to Primary Resistance of Anti-PD1 Therapy in Hepatocellular Carcinoma	10.3389/fimmu.2022.876048	Article	2022	Not experimentally demonstrating a direct association between the ICI effect on changes in the levels of miRNAs or vice versa
Overcoming anti-cancer drug resistance via restoration of tumor suppressor gene function	10.1016/j.drup.2021.100770	Review	2021	Not an original study
Overcoming resistance to PD-1/PD-L1 inhibitors in esophageal cancer	10.3389/fonc.2022.955163	Review	2022	Not an original study
P1.15-02 Plasma microRNAs Modulation in Advanced NSCLC Patients Receiving Single Agent Immune Checkpoint Inhibitors	10.1016/j.jtho.2022.07.198	Conference Abstract	2022	Not an original study
P82.02 Stereotactic Ablative Radiotherapy with Nivolumab for Early-Stage Operable Non-Small Cell Lung Cancer: a phase 2 study	10.1016/j.jtho.2021.01.1193	Conference Abstract	2021	Not an original study
Pan-Cancer Analyses Identify the CTC1-STN1-TEN1 Complex as a Protective Factor and Predictive Biomarker for Immune Checkpoint Blockade in Cancer	10.3389/fgene.2022.859617	Article	2022	Not experimentally demonstrating a direct association between the ICI effect on changes in the levels of miRNAs or vice versa
Pan-cancer analyses reveal regulation and clinical outcome association of the shelterin complex in cancer	10.1093/bib/bbaa441	Article	2021	Not experimentally demonstrating a direct association between the ICI effect on changes in the levels of miRNAs or vice versa
Pan-cancer landscape of CD274 (PD-L1) rearrangements in 283,050 patient samples, its correlation with PD-L1 protein expression, and immunotherapy response	10.1136/jitc-2021-003550	Article	2021	Not focused on miRNAs
Pathogenetic features and current management of glioblastoma	10.3390/cancers13040856	Review	2021	Not an original study
PD-1 inhibitor inducing exosomal miR-34a-5p expression mediates the cross talk between cardiomyocyte and macrophage in immune checkpoint inhibitor-related cardiac dysfunction	10.1136/jitc-2020-001293	Article	2020	Article included in qualitative synthesis
PD-1/PD-L1-dependent immune response in colorectal cancer	10.1002/jcp.29494	Review	2020	Not an original study
PD-L1 regulation revisited: impact on immunotherapeutic strategies	10.1016/j.molmed.2021.06.005	Review	2021	Not an original study
Personalized Immunotherapy in Colorectal Cancers: Where Do We Stand?	10.3389/fonc.2021.769305	Review	2021	Not an original study
Pertuzumab and trastuzumab for HER2-positive, metastatic biliary tract cancer (MyPathway): a multicentre, open-label, phase 2a, multiple basket study	10.1016/S1470-2045(21)00336-3	Article	2021	Not focused on miRNAs
Phase 2 study of pembrolizumab in patients with advanced rare cancers	10.1136/jitc-2019-000347	Article	2020	Not focused on miRNAs
Phase I/II study of the LAG-3 inhibitor ieramilimab (LAG525) ± anti-PD-1 spartalizumab (PDR001) in patients with advanced malignancies	10.1136/jitc-2021-003776	Article	2022	Not focused on miRNAs
Pivotal role of long non-coding ribonucleic acid-X-inactive specific transcript in regulating immune checkpoint programmed death ligand 1 through a shared pathway between miR-194-5p and miR-155-5p in hepatocellular carcinoma		Article	2020	Not experimentally demonstrating a direct association between the ICI effect on changes in the levels of miRNAs or vice versa
Plasma exosome microRNA-155 expression in patients with metastatic renal cell carcinoma treated with immune checkpoint inhibitors: A potential biomarker of response to systemic therapy	10.1200/JCO.2021.39.15_suppl.4570	Conference Abstract	2021	Not an original study
Plasma exosome microRNA-155-3p expression in patients with metastatic renal cell carcinoma treated with immune checkpoint inhibitors: Potential biomarker of response to systemic therapy	10.1016/j.annonc.2021.08.089	Conference Abstract	2021	Not an original study
Plasma-Based microRNA Expression Analysis in Advanced Stage NSCLC Patients Treated with Nivolumab	10.3390/cancers14194739	Article	2022	Not experimentally demonstrating a direct association between the ICI effect on changes in the levels of miRNAs or vice versa

Potentiating therapeutic effects of epidermal growth factor receptor inhibition in triple-negative breast cancer	10.3390/ph14060589	Review	2021	Not an original study
Predictive biomarkers for systemic therapy of hepatocellular carcinoma	10.1080/14737159.2021.1987217	Review	2021	Not an original study
Predictive role of plasmatic biomarkers in advanced non-small cell lung cancer treated by nivolumab	10.1080/2162402X.2018.1452581	Article	2018	Not experimentally demonstrating a direct association between the ICI effect on changes in the levels of miRNAs or vice versa
Profilin 1 protein and its implications for cancers	10.46883/ONC.2021.3507.0402	Review	2021	Not an original study
Prognostic and predictive biomarkers in gliomas	10.3390/ijms221910373	Review	2021	Not an original study
Prognostic and predictive biomarkers in stage iii melanoma: Current insights and clinical implications	10.3390/ijms22094561	Review	2021	Not an original study
Prognostic Characteristics of Immune-Related Genes and the Related Regulatory Axis in Patients With Stage N+M0 Breast Cancer	10.3389/fonc.2022.878219	Article	2022	Not experimentally demonstrating a direct association between the ICI effect on changes in the levels of miRNAs or vice versa
Prognostic Ferroptosis-Related lncRNA Signatures Associated With Immunotherapy and Chemotherapy Responses in Patients With Stomach Cancer	10.3389/fgene.2021.798612	Article	2021	Not experimentally demonstrating a direct association between the ICI effect on changes in the levels of miRNAs or vice versa
Prophylactic In Vivo Hematopoietic Stem Cell Gene Therapy with an Immune Checkpoint Inhibitor Reverses Tumor Growth in Syngeneic Mouse Tumor Models	10.1158/0008-5472.CAN-19-1044	Article	2020	Not experimentally demonstrating a direct association between the ICI effect on changes in the levels of miRNAs or vice versa
Prostaglandin E2 Receptor 4 (EP4) as a Therapeutic Target to Impede Breast Cancer-Associated Angiogenesis and Lymphangiogenesis	10.3390/cancers13050942	Review	2021	Not an original study
Proteogenomic characterization of human colon cancer reveals new therapeutic opportunities	10.1158/1538-7445.AM2019-LB-006	Meeting Abstract	2019	Not an original study
Quadruple negative breast cancer	10.1007/s12282-020-01047-6	Review	2020	Not an original study
Quantifying the contribution of transcription factor activity, mutations and microRNAs to CD274 expression in cancer patients	10.1038/s41598-022-08356-0	Article	2022	Not experimentally demonstrating a direct association between the ICI effect on changes in the levels of miRNAs or vice versa
Racial disparity in prostate cancer in the African American population with actionable ideas and novel immunotherapies	10.1002/cnr2.1340	Review	2021	Not an original study
Recent Advances in Glioma Cancer Treatment: Conventional and Epigenetic Realms	10.3390/vaccines10091448	Review	2022	Not an original study
Recent advances in pancreatic cancer: Novel prognostic biomarkers and targeted therapy—a review of the literature	10.3390/biom11101469	Review	2021	Not an original study
Recent advances in the role of Th17/Treg cells in tumor immunity and tumor therapy	10.1007/s12026-021-09211-6	Review	2021	Not an original study
Recurrent myocarditis induced by immune-checkpoint inhibitor treatment is accompanied by persistent inflammatory markers despite immunosuppressive treatment	10.1200/PO.20.00370	Article	2021	Not focused on miRNAs
Refining immunotherapeutic approaches to high-risk neuroblastoma based on tumor genomic profiles	10.1002/1878-0261.12880	Note	2021	Not an original study
Regulation and function of autophagy in pancreatic cancer	10.1080/15548627.2020.1847462	Review	2021	Not an original study
Regulation of CD47 expression in cancer cells		Review	2020	Not an original study
Regulation of PD-1/PD-L1 Pathway in Cancer by Noncoding RNAs		Review	2020	Not an original study
Regulatory T cells and their associated factors in hepatocellular carcinoma development and therapy	10.3748/wjg.v28.i27.3346	Review	2022	Not an original study
Relationship Between CNVs and Immune Cells Infiltration in Gastric Tumor Microenvironment	10.3389/fgene.2022.869967	Article	2022	Not focused on miRNAs
Resistance of Lenvatinib in Hepatocellular Carcinoma	10.2174/1568009622666220428111327	Review	2022	Not an original study
Restoration of miR-340 controls pancreatic cancer cell CD47 expression to promote macrophage phagocytosis and enhance antitumor immunity		Article	2020	Article included in qualitative synthesis
Resveratrol as an enhancer of apoptosis in cancer: A mechanistic review	10.2174/1871520620666201020160348	Review	2021	Not an original study
Reversal of resistance to anti-cd20 antibody therapies: Targeting intracellular resistant factors	10.1615/CritRevOncog.2020036109	Review	2020	Not an original study
Review article: systemic treatment of hepatocellular carcinoma	10.1111/apt.14913	Review	2018	Not an original study
Risk Factors and Biomarkers for Immune-Related Adverse Events: A Practical Guide to Identifying High-Risk Patients and Rechallenging Immune Checkpoint Inhibitors	10.3389/fimmu.2022.779691	Review	2022	Not an original study
Risk of reactivation of occult hepatitis B during immunotherapy in cancer treatment: Myth, reality or new horizons?	10.2217/fon-2020-1196	Note	2021	Not an original study
RNA N6-methyladenosine: a new player in autophagy-mediated anti-cancer drug resistance	10.1038/s41416-021-01314-z	Note	2021	Not an original study
Role of Abdominal and Pelvic CT Scans in Diagnosis of Patients with Immunotherapy-Induced Colitis	10.36401/JIPO-21-21	Article	2022	Not focused on miRNAs
Role of Epigenetic Regulation in Plasticity of Tumor Immune Microenvironment	10.3389/fimmu.2021.640369	Review	2021	Not an original study
Role of Exosomes in Immunotherapy of Hepatocellular Carcinoma	10.3390/cancers14164036	Review	2022	Not an original study
Role of miRNAs in immune responses and immunotherapy in cancer	10.1002/gcc.22725	Review	2019	Not an original study
Role of tumor microenvironment in the regulation of PD-L1: A novel role in resistance to cancer immunotherapy		Review	2020	Not an original study
Roles of Cadherin2 in Thyroid Cancer	10.3389/fonc.2022.804287	Article	2022	Not experimentally demonstrating a direct association between the ICI effect on changes in the levels of miRNAs or vice versa
Rs10754339 (A/G) in 3'UTR of B7-H4 regulated by miR-506-3p prevents breast cancer progression		Conference Abstract	2018	Not an original study
Safety and activity of microRNA-loaded minicells in patients with recurrent malignant pleural mesothelioma: a first-in-man, phase 1, open-label, dose-escalation study	10.1016/S1470-2045(17)30621-6	Article	2017	Not experimentally demonstrating a direct association between the ICI effect on changes in the levels of miRNAs or vice versa
Serum exosomal miR-16-5p functions as a tumor inhibitor and a new biomarker for PD-L1 inhibitor-dependent immunotherapy in lung adenocarcinoma by regulating PD-L1 expression	10.1002/cam4.4638	Article	2022	Article included in qualitative synthesis
SETD2-mediated loss of miR-339 promotes tumor PD-L1 expression and attenuates antitumor immunity in renal cell carcinoma		Conference Abstract	2019	Not an original study
Shifting gears in precision oncology—challenges and opportunities of integrative data analysis	10.3390/biom11091310	Review	2021	Not an original study
Shooting at moving and hidden targets—tumour cell plasticity and the notch signalling pathway in head and neck squamous cell carcinomas	10.3390/cancers13246219	Review	2021	Not an original study
Signal Pathway of Estrogen and Estrogen Receptor in the Development of Thyroid Cancer	10.3389/fonc.2021.593479	Review	2021	Not an original study
Silencing EGFR-upregulated expression of CD55 and CD59 activates the complement system and sensitizes lung cancer to checkpoint blockade	10.1038/s43018-022-00444-4	Article	2022	Not experimentally demonstrating a direct association between the ICI effect on changes in the levels of miRNAs or vice versa
Single-cell analysis of a tumor-derived exosome signature correlates with prognosis and immunotherapy response		Article	2021	Not focused on miRNAs
Small Extracellular Vesicles: A Novel Avenue for Cancer Management	10.3389/fonc.2021.638357	Review	2021	Not an original study
Soft tissue sarcoma: An insight on biomarkers at molecular, metabolic and cellular level	10.3390/cancers13123044	Review	2021	Not an original study
Soluble Biomarkers with Prognostic and Predictive Value in Advanced Non-Small Cell Lung Cancer Treated with Immunotherapy	10.3390/cancers13174280	Review	2021	Not an original study
Soluble PD-1: Predictive, Prognostic, and Therapeutic Value for Cancer Immunotherapy	10.3389/fimmu.2020.587460	Review	2020	Not an original study
Sos2 comes to the fore: Differential functionalities in physiology and pathology	10.3390/ijms22126613	Review	2021	Not an original study
Sotorasib for previously treated colorectal cancers with KRAS(G12C) mutation (CodeBreaK100): a prespecified analysis of a single-arm, phase 2 trial	10.1016/S1470-2045(21)00605-7	Article	2022	Not focused neither on miRNA nor on ICIs
Spatial and phenotypic immune profiling of metastatic colon cancer		Article	2018	Not focused on miRNAs
Special issue: Resistance mechanisms to cancer immunotherapy	10.1016/j.semcancer.2020.01.014	Editorial	2020	Not an original study
Spectrum of Immune Checkpoint Inhibitor Anemias: Results From a Single Center, Early-Phase Clinical Trials Case Series Experience	10.14740/jh1006	Article	2022	Not focused on miRNAs
Stem cells as therapeutic targets in colorectal cancer	10.2217/pme-2020-0099	Review	2021	Not an original study
Suppression of microRNA activity amplifies IFN-γ-induced macrophage activation and promotes anti-tumour immunity		Article	2016	Not experimentally demonstrating a direct association between the ICI effect on changes in the levels of miRNAs or vice versa
Surface Heparinization of a Magnesium-Based Alloy: A Comparison Study of Aminopropyltriethoxysilane (APTES) and Polyamidoamine (PAMAM) Dendrimers	10.3390/jfb13040296	Article	2022	Not focused neither on miRNA nor on ICIs
Systematic analysis of berberine-induced signaling pathway between miRNA clusters and mRNAs and identification of mir-99a similar to 125b cluster function by seed-targeting inhibitors in multiple myeloma ce	10.1080/15476286.2015.1017219	Systematic revision	2015	Not an original study
Systemic Therapy and Sequencing Options in Advanced Hepatocellular Carcinoma: A Systematic Review and Network Meta-analysis	10.1001/jamaoncol.2020.4930	Systematic revision	2020	Not an original study
Targeted drug delivery system for ovarian cancer microenvironment: Improving the effects of immunotherapy	10.3389/fimmu.2022.1035997	Review	2022	Not an original study
Targeted nano-drug delivery of colchicine against colon cancer cells by means of mesoporous silica nanoparticles	10.3390/cancers12010144	Article	2020	Not experimentally demonstrating a direct association between the ICI effect on changes in the levels of miRNAs or vice versa
Targeted therapy and personalized medicine in hepatocellular carcinoma: drug resistance, mechanisms, and treatment strategies	10.2147/JHC.S106529	Review	2017	Not an original study
Targeted treatment of head and neck (Pre)cancer: Preclinical target identification and development of novel therapeutic applications	10.3390/cancers13112774	Review	2021	Not an original study
Targeting Cancer Stem Cells through Epigenetic Modulation of Interferon Response	10.3390/jpm12040556	Review	2022	Not an original study
Targeting KRAS: Crossroads of Signaling and Immune Inhibition	10.36401/JIPO-22-5	Review	2022	Not an original study
Targeting Myeloid-Derived Suppressor Cells to Enhance the Antitumor Efficacy of Immune Checkpoint Blockade Therapy	10.3389/fimmu.2021.754196	Review	2021	Not an original study
Targeting non-coding RNAs to overcome cancer therapy resistance	10.1038/s41392-022-00975-3	Review	2022	Not an original study
Targeting nuclear acid-mediated immunity in cancer immune checkpoint inhibitor therapies	10.1038/s41392-020-00347-9	Review	2020	Not an original study
Targeting PD-1/PD-L1 axis as new horizon for ovarian cancer therapy	10.1016/j.lfs.2022.120827	Review	2022	Not an original study
Targeting T Cell Subtypes for NAFLD and NAFLD-Related HCC Treatment: An Opinion	10.3389/fmed.2021.789859	Review	2021	Not an original study
Targeting tumor-associated macrophages to synergize tumor immunotherapy	10.1038/s41392-021-00484-9	Review	2021	Not an original study
Tertiary lymphoid structure signatures are associated with survival and immunotherapy response in muscle-invasive bladder cancer	10.1080/2162402X.2021.1915574	Article	2021	Not experimentally demonstrating a direct association between the ICI effect on changes in the levels of miRNAs or vice versa
Thalidomide suppresses angiogenesis and immune evasion via lncRNA FGD5-AS1/miR-454-3p/ZEB1 axis-mediated VEGFA expression and PD-1/PD-L1 checkpoint in NSCLC	10.1016/j.cbi.2021.109652	Article	2021	Not experimentally demonstrating a direct association between the ICI effect on changes in the levels of miRNAs or vice versa
The 150 most important questions in cancer research and clinical oncology series: questions 57-66 : Edited by Chinese Journal of Cancer	10.1186/s40880-017-0249-9	Review	2017	Not an original study
The biological complexity of urothelial carcinoma: Insights into carcinogenesis, targets and biomarkers of response to therapeutic approaches	10.1016/j.semcancer.2015.08.006	Review	2015	Not an original study
The CDK1-Related lncRNA and CXCL8 Mediated Immune Resistance in Lung Adenocarcinoma	10.3390/cells11172688	Article	2022	Not experimentally demonstrating a direct association between the ICI effect on changes in the levels of miRNAs or vice versa
The Chemokine System in Oncogenic Pathways Driven by Viruses: Perspectives for Cancer Immunotherapy	10.3390/cancers14030848	Review	2022	Not an original study
The circular RNA circDLG1 promotes gastric cancer progression and anti-PD-1 resistance through the regulation of CXCL12 by sponging miR-141-3p	10.1186/s12943-021-01475-8	Article	2021	Not experimentally demonstrating a direct association between the ICI effect on changes in the levels of miRNAs or vice versa
The clinical outcome and risk factors analysis of immune checkpoint inhibitor-based treatment in lung adenocarcinoma patients with brain metastases	10.21037/tlcr-22-260	Article	2022	Not experimentally demonstrating a direct association between the ICI effect on changes in the levels of miRNAs or vice versa
The Combination of Radiotherapy With Immunotherapy and Potential Predictive Biomarkers for Treatment of Non-Small Cell Lung Cancer Patients	10.3389/fimmu.2021.723609	Review	2021	Not an original study
The crosstalk between tumor cells and the microenvironment in hepatocellular carcinoma: The role of exosomal micrornas and their clinical implications	10.3390/cancers12040823	Review	2020	Not an original study
The crucial roles of long noncoding RNA SNHGs in lung cancer	10.1007/s12094-022-02909-5	Review	2022	Not an original study
The emerging role of miRNA in the perturbation of tumor immune microenvironment in chemoresistance: Therapeutic implications	10.1016/j.semcdb.2021.04.001	Review	2022	Not an original study
The Emerging Role of T-Cell Immunoglobulin Mucin-3 in Breast Cancer: A Promising Target For Immunotherapy	10.3389/fonc.2021.723238	Review	2021	Not an original study
The enigma of Kaposi sarcoma management	10.1136/jim-2021-MW.52	Conference Abstract	2021	Not an original study
The expression pattern of Immune checkpoints after chemo/radiotherapy in the tumor microenvironment	10.3389/fimmu.2022.938063	Review	2022	Not an original study
The Extrinsic and Intrinsic Roles of PD-L1 and Its Receptor PD-1: Implications for Immunotherapy Treatment	10.3389/fimmu.2020.568931	Review	2020	Not an original study
The generation of PD-L1 and PD-L2 in cancer cells: From nuclear chromatin reorganization to extracellular presentation		Review	2022	Not an original study
The immuno-therapeutic potential of miR-2392 in patients with hepatocellular carcinoma	10.1111/liv.15395	Letter	2022	Not an original study
The importance of establishing genetic phase in clinical medicine	10.1111/iji.12567	Review	2022	Not an original study
The importance of immune checkpoints in immune monitoring: A future paradigm shift in the treatment of cancer	10.1016/j.biopha.2021.112516	Review	2022	Not an original study
The influence of tumor microenvironment on immune escape of melanoma	10.3390/ijms21218359	Review	2020	Not an original study
The Interplay between MicroRNAs and the Components of the Tumor Microenvironment in B-Cell Malignancies		Review	2020	Not an original study
The let-7 family of microRNAs suppresses immune evasion in head and neck squamous cell carcinoma by promoting PD-L1 degradation	10.1186/s12964-019-0490-8	Article	2019	Article included in qualitative synthesis
The miR-199a-5p/PD-L1 axis regulates cell proliferation, migration and invasion in follicular thyroid carcinoma	10.1186/s12885-022-09838-0	Article	2022	Not experimentally demonstrating a direct association between the ICI effect on changes in the levels of miRNAs or vice versa
The miR-200 family of microRNAs: Fine tuners of epithelial-mesenchymal transition and circulating cancer biomarkers	10.3390/cancers13235874	Review	2021	Not an original study
The miR-25-93-106b cluster regulates tumor metastasis and immune evasion via modulation of CXCL12 and PD-L1	10.18632/oncotarget.15450	Article	2017	Not experimentally demonstrating a direct association between the ICI effect on changes in the levels of miRNAs or vice versa
The miRNA expression profile of inflammatory tumors reveals a unique immune cell profile and potential companion targets for checkpoint blockade immunotherapy		Abstract meeting	2019	Not an original study
The miRNA Profile of Inflammatory Colorectal Tumors Identify TGF-β as a Companion Target for Checkpoint Blockade Immunotherapy	10.3389/fcell.2021.754507	Article	2021	Not experimentally demonstrating a direct association between the ICI effect on changes in the levels of miRNAs or vice versa
The Molecular Basis and Therapeutic Aspects of Cisplatin Resistance in Oral Squamous Cell Carcinoma	10.3389/fonc.2021.761379	Review	2021	Not an original study
The nivolumab/cabozantinib combination and the future of doublets in advanced renal cell cancer treatment	10.1177/10781552211027942	Letter	2021	Not an original study
The origin of gastric cancer stem cells and their effects on gastric cancer: Novel therapeutic targets for gastric cancer	10.3389/fonc.2022.960539	Review	2022	Not an original study
The pleiotropic roles of circular and long noncoding RNAs in cutaneous melanoma	10.1002/1878-0261.13034	Review	2022	Not an original study
The positive and negative immunoregulatory role of b7 family: Promising novel targets in gastric cancer treatment	10.3390/ijms221910719	Review	2021	Not an original study
The Potential Regulatory Roles of Circular RNAs in Tumor Immunology and Immunotherapy		Review	2021	Not an original study
The Prognostic Significance and Potential Mechanism of Prolyl 3-Hydroxylase 1 in Hepatocellular Carcinoma	10.1155/2022/7854297	Article	2022	Not focused on ICIs
The prognostic value and immunological role of angiogenesis-related patterns in colon adenocarcinoma	10.3389/fonc.2022.1003440	Article	2022	Not focused on miRNAs
The progress of immune checkpoint therapy in primary liver cancer	10.1016/j.bbcan.2021.188638	Review	2021	Not an original study
The Promise of Targeting Hypoxia to Improve Cancer Immunotherapy: Mirage or Reality?	10.3389/fimmu.2022.880810	Review	2022	Not an original study
The Regulatory Effects of MicroRNAs on Tumor Immunity	10.1155/2022/2121993	Review	2022	Not an original study
The regulatory role of long non- coding RNAs as a novel controller of immune response against cancer cells	10.1007/s11033-022-07947-4	Review	2022	Not an original study
The role and therapeutic targeting of JAK/STAT signaling in glioblastoma	10.3390/cancers13030437	Review	2021	Not an original study
The role of B7-H3 in tumors and its potential in clinical application	10.1016/j.intimp.2021.108153	Review	2021	Not an original study
The Role of Circular RNAs in the Drug Resistance of Cancers	10.3389/fonc.2021.790589	Review	2022	Not an original study
The role of ferroptosis in melanoma	10.1111/pcmr.13009	Review	2022	Not an original study
The Role of Genomics and Proteomics in Lung Cancer Early Detection and Treatment	10.3390/cancers14205144	Review	2022	Not an original study
The role of host molecules in communication with the resident and pathogenic microbiota: A review	10.1016/j.medmic.2020.100005	Review	2020	Not an original study
The Role of NcRNAs to Regulate Immune Checkpoints in Cancer	10.3389/fimmu.2022.853480	Review	2022	Not an original study
The role of tumor associated macrophages (Tams) in cancer pro-gression, chemoresistance, angiogenesis and metastasis-current status	10.2174/0929867328666210720143721	Review	2021	Not an original study
The Roles of MiRNAs (MicroRNAs) in Melanoma Immunotherapy	10.3390/ijms232314775	Review	2022	Not an original study
The Significance of Tumor Microenvironment Score for Breast Cancer Patients	10.1155/2022/5673810	Article	2022	Not experimentally demonstrating a direct association between the ICI effect on changes in the levels of miRNAs or vice versa
Therapeutics Targeting FGF Signaling Network in Human Diseases	10.1016/j.tips.2016.10.003	Review	2016	Not an original study
Think global but act local: Tuning the dendritic cell response in cancer	10.1016/j.biocel.2022.106227	Short Survey	2022	Not an original study
Thirty years of Epstein-Barr virus-associated gastric carcinoma	10.1007/s00428-019-02724-4	Review	2020	Not an original study
Tim-3 promotes tumor-promoting M2 macrophage polarization by binding to STAT1 and suppressing the STAT1-miR-155 signaling axis	10.1080/2162402X.2016.1211219	Article	2016	Not experimentally demonstrating a direct association between the ICI effect on changes in the levels of miRNAs or vice versa
Tissue- and liquid-based biomarkers in prostate cancer precision medicine	10.3390/jpm11070664	Review	2021	Not an original study
Transcriptional regulation of cancer immune checkpoints: Emerging strategies for immunotherapy	10.3390/vaccines8040735	Review	2020	Not an original study
Translational Implications of Dysregulated Pathways and microRNA Regulation in Quadruple-Negative Breast Cancer	10.3390/biomedicines10020366	Review	2022	Not an original study
Translational Modeling Identifies Synergy between Nanoparticle-Delivered miRNA-22 and Standard-of-Care Drugs in Triple-Negative Breast Cancer	10.1007/s11095-022-03176-3	Article	2022	Not experimentally demonstrating a direct association between the ICI effect on changes in the levels of miRNAs or vice versa
Tumor Microenvironment Analysis Identified Subtypes Associated With the Prognosis and the Tumor Response to Immunotherapy in Bladder Cancer	10.3389/fgene.2021.551605	Article	2021	Not experimentally demonstrating a direct association between the ICI effect on changes in the levels of miRNAs or vice versa
Tumor Secretome to Adoptive Cellular Immunotherapy: Reduce Me Before I Make You My Partner	10.3389/fimmu.2021.717850	Review	2021	Not an original study
Tumor-derived microRNAs induce myeloid suppressor cells and predict immunotherapy resistance in melanoma	10.1172/JCI98060	Article	2018	Not experimentally demonstrating a direct association between the ICI effect on changes in the levels of miRNAs or vice versa
Tumor-Intrinsic PD-L1 Exerts an Oncogenic Function through the Activation of the Wnt/β-Catenin Pathway in Human Non-Small Cell Lung Cancer	10.3390/ijms231911031	Article	2022	Not experimentally demonstrating a direct association between the ICI effect on changes in the levels of miRNAs or vice versa
Tumor-intrinsic SIRPA promotes sensitivity to checkpoint inhibition immunotherapy in melanoma	10.1016/j.ccell.2022.10.012	Review	2022	Not an original study
Tumor-Secreted Extracellular Vesicles Regulate T-Cell Costimulation and Can Be Manipulated To Induce Tumor-Specific T-Cell Responses	10.1053/j.gastro.2021.04.036	Article	2021	Article included in qualitative synthesis
Tumor-Specific and Tumor-Agnostic Molecular Signatures Associated With Response to Immune Checkpoint Inhibitors	10.1200/PO.21.00008	Article	2021	Not focused on miRNAs
Tumour evolution and seed and soil mechanism in pancreatic metastases of renal cell carcinoma	10.3390/cancers13061342	Review	2021	Not an original study
Twentieth Anniversary of Molecular Cancer Therapeutics: A Vision for the Future	10.1158/1535-7163.MCT-21-0771	Editorial Material	2021	Not an original study
Ulcerated Cutaneous Melanoma: A Review of the Clinical, Histologic, and Molecular Features Associated with a Clinically Aggressive Histologic Phenotype	10.2147/CCID.S372287	Review	2022	Not an original study
Uncovering the Subtype-Specific Molecular Characteristics of Breast Cancer by Multiomics Analysis of Prognosis-Associated Genes, Driver Genes, Signaling Pathways, and Immune Activity	10.3389/fcell.2021.689028	Article	2021	Not focused neither on miRNA nor on ICIs
Unique features of hepatitis b virus-related hepatocellular carcinoma in pathogenesis and clinical significance	10.3390/cancers13102454	Review	2021	Not an original study
Unveiling Novel Therapeutic Drug Targets and Prognostic Markers of Triple Negative Breast Cancer	10.2174/1568009621666210908113010	Review	2021	Not an original study
Upregulating hsa-miR-128a Increased the Effects of Pembrolizumab on Laryngeal Cancer Cells via the p53 Pathway	10.1155/2021/2342784	Article	2021	Article included in qualitative synthesis
Utomilumab in Patients With Immune Checkpoint Inhibitor-Refractory Melanoma and Non-Small-Cell Lung Cancer	10.3389/fimmu.2022.897991	Article	2022	Not focused on miRNAs
Virally programmed extracellular vesicles sensitize cancer cells to oncolytic virus and small molecule therapy	10.1038/s41467-022-29526-8	Article	2022	Not experimentally demonstrating a direct association between the ICI effect on changes in the levels of miRNAs or vice versa
What Do We Have to Know about PD-L1 Expression in Prostate Cancer? A Systematic Literature Review. Part 5: Epigenetic Regulation of PD-L1	10.3390/ijms222212314	Review	2021	Not an original study
What Is Known about Theragnostic Strategies in Colorectal Cancer	10.3390/biomedicines9020140	Review	2021	Not an original study

**Win or loss? Combination therapy does improve the oncolytic virus therapy to pancreatic cancer**	10.1186/s12935-022-02583-1	Review	2022	Not an original study
**World Cancer Day 2021 - Perspectives in Pediatric and Adult Neuro-Oncology**	10.3389/fonc.2021.659800	Review	2021	Not an original study
**XIST/miR-34a-5p/PDL1 axis regulated the development of lung cancer cells and the immune function of CD8(+) T cells**	10.1080/10799893.2021.2019274	Article	2022	Not experimentally demonstrating a direct association between the ICI effect on changes in the levels of miRNAs or vice versa
**ZCCHC17 Served as a Predictive Biomarker for Prognosis and Immunotherapy in Hepatocellular Carcinoma**	10.3389/fonc.2021.799566	Article	2021	Not focused on miRNAs
